# Anticholinesterase Activity of Selected Medicinal Plants from Navarra Region of Spain and a Detailed Phytochemical Investigation of *Origanum vulgare* L. ssp. *vulgare*

**DOI:** 10.3390/molecules27207100

**Published:** 2022-10-20

**Authors:** María Pilar de Torre, Rita Yolanda Cavero, María Isabel Calvo

**Affiliations:** 1 Department of Pharmaceutical Technology and Chemical, Faculty of Pharmacy and Nutrition, University of Navarra, Irunlarrea s/n, 31008 Pamplona, Spain; 2 Department of Environmental Biology, Faculty of Sciences, University of Navarra, Irunlarrea s/n, 31008 Pamplona, Spain; 3 Instituto de Investigación Sanitaria de Navarra (IDISNA), 31008 Pamplona, Spain

**Keywords:** Alzheimer, ethnopharmacology, oregano, chromatography, phenolic compounds, syringic acids

## Abstract

Alzheimer’s disease is a neurodegenerative disease characterized by progressive memory loss and cognitive impairment due to a severe loss of cholinergic neurons in specific brain areas. It is the most common type of dementia in the aging population. Although many anti-acetylcholinesterase (AChE) drugs are already available on the market, their performance sometimes yields unexpected results. For this reason, research works are ongoing to find potential anti-AChE agents both from natural and synthetic sources. In this study, 90 extracts from 30 native and naturalized medicinal plants are tested by TLC and Ellman’s colorimetric assay at 250, 125 and 62.5 μg/mL in order to determine the inhibitory effect on AChE. In total, 21 out of 90 extracts show high anti-AChE activity (75–100% inhibition) in a dose-dependent manner. Among them, ethanolic extract from aerial parts of *O. vulgare* ssp. *vulgare* shows an IC50 value 7.7 times lower than galantamine. This research also establishes the chemical profile of oregano extract by TLC, HPLC-DAD and LC-MS, and twenty-three compounds are identified and quantified. Dihydroxycinnamic acids and flavonoids are the most abundant ones (56.90 and 25.94%, respectively). Finally, total phenolic compounds and antioxidant properties are quantified by colorimetric methods. The total phenolic content is 207.64 ± 0.69 µg/mg of extract. The antioxidant activity is measured against two radicals, DPPH and ABTS. In both assays, the oregano extract shows high activity. The Pearson correlation matrix shows the relationship between syringic acids, a type of dihydroxybenzoic acid, and anti-AChE (r^2^ = −0.9864) and antioxidant activity (r^2^ = 0.9409 and 0.9976). In conclusion, the results of this study demonstrate promising potential new uses of these medicinal herbs for the treatment of Alzheimer’s. *Origanum vulgare* ssp. *vulgare* and syringic acids, which have anti-AChE activity and beneficial antioxidant capacity, can be highlighted as potential candidates for the development of drugs for the treatment of Alzheimer’s disease and other diseases characterized by a cholinergic deficit.

## 1. Introduction

Alzheimer’s disease (AD) is the most common cause of dementia. This chronic neurodegenerative disease develops slowly and progressively by causing deterioration of intellectual capacity in the following Wernicke areas: learning and memory, language abilities, reading and writing, praxis, interaction with the environment and personality changes. Early detection of the disease is important because, as of now, medicine cannot reverse degeneration but can only delay the neurodegenerative progression. Risk factors to develop AD include both genetic (gene ApoE4) and environmental factors (age, depression, metabolic syndrome: HTA, diabetes and hyperlipidaemia) [1].

Since the 1970–1980s, science has focused on “The Cholinergic hypothesis of AD” because of the highly consistent findings on the alteration of some selective neurotransmitter systems in patients with AD. A presynaptic reduction of acetylcholine (ATCh) was found in patients with AD and amongst other Alzheimer’s treatments, inhibitors of acetylcholinesterase, that increase this neurotransmitter in the neocortical synaptic space, are the most common ones. The most important ones are donepezile, galantamine and rivastigmine, especially used in the early stages of the disease [2], having hepatotoxicity and gastrointestinal disorders as side effects [3].

As aging and oxidative stress (production of reactive oxygen species—ROS) are involved in AD [4], antioxidants might also be potentially helpful in Alzheimer’s treatment.

The ethnopharmacological study is one of the best ways for drug discovery and development. This research is mainly carried out by academic institutions rather than by the pharmaceutical industry. In the first steps of investigations, traditional use and preparation techniques of medicinal plants can be used as a guide for the extraction methods and in vitro pharmacological screening. Later on, the industry can conduct in vivo targeted screenings and clinical trials [5].

In northern Spain, where the province of Navarra is located, there is a great diversity of native and naturalized medicinal plants. In 2003, our research group started an ethnopharmacological investigation that continues to the present day. The high number of plants collected to date has allowed the publication of several manuscripts for various affections, neurological and mental disorders among them [6]. The aim of the current study is the analysis of 90 extracts obtained from 38 plant species used in Navarra for the nervous system, followed by the selection of the most active ones for chemical characterization.

## 2. Results and Discussion

### 2.1. Antiacethylcholinesterase Activity

In the last two decades, the mechanism of inhibition of AChE has acquired high importance in treating AD symptoms from a clinical point of view. Some extracts and phytochemicals have shown this activity [7]. Several methods have been described for the determination of AChE inhibitory activity, such as colorimetric methods using Ellman’s reagent or Fast Blue B salt reagent, fluorometric methods or HPLC online detection. Ellman´s method, which is based on the determination of the amount of thiocholine released when acetylthiocholine is hydrolyzed by AChE, is the most widely employed method because it is simple and gives quick access to information in plant extracts [8].

In order to select plant extracts with high AChE inhibitory activity, in this study, 90 ethanolic and aqueous extracts of 38 medicinal plants collected in Navarra (Spain) were analyzed. These medicinal plants, belonging to nine botanical families (*Asteraceae*, *Lamiaceae*, *Crassulaceae*, *Equisetaceae*, *Euphorbiaceae*, *Lythraceae*, *Papaveraceae*, *Primulaceae* and *Verbenaceae*), showed high antioxidant activity in previous researches of our group [9,10].

Qualitative screening by TLC showed that 20 out of the 90 extracts were inactive at doses of 0.20 mg. Since the crude extracts may sometimes yield false positive or negative results in the TLC assay, a quantitative microplate assay was performed. In total, 20 extracts showed an inhibition rate below 10% at a dose of 250 μg/mL (Table 1). A summary of screening studies of these extracts is provided in Table 1, alphabetically ordered by family, showing scientific name, botanical part, extraction solvent, yield of extraction, percentage of inhibition and concentration at which 50% of the enzyme is inhibited. Seventy extracts showed inhibitory activity towards acetylcholinesterase at a concentration of 250 μg/mL, 21 of them with high activity (75–100% inhibition), 34 with moderate activity (50–75% inhibition) and 15 with low activity (10–50% inhibition) (Table 1).

The screening of 30 extracts from 11 medicinal plants of the *Asteraceae* family revealed that two of them exhibited very strong activity with inhibition percentages higher than 75%: the aqueous extract from the leaves of *Tussilago farfara* (80.25 ± 13.78%) and ethanolic extracts from the inflorescence of *Santolina chamaecyparissus* (75.82 ± 6.69%) at 250 μg/mL. Ethyl acetate extracts of *T. dubius* and *T. farfara* have been described as potent inhibitors of acetylcholinesterase and butyrylcholinesterase [11].

In relation to *S. chamaecyparissus*, only the essential oil obtained by steam distillation has been described as a control agent against termites due to this activity [12]. It has also been described this activity in the essential oil of other species from *Santolina*, such as *S. impresa* [13] and *S. semidentata* [14]. However, it is important to highlight that the ethanolic extract of *S. chamaecyparissus* has a different chemical composition compared to the essential oil and contains non-volatile compounds, which could be a source of new bioactive compounds.

*Lamiaceae* species have been reported to possess a wide range of biological activity and a diversity of phytochemicals. This botanical family is rich in essential oils, hydroxycinnamic acids and flavonoids as active constituents, which significantly contribute to its neuroprotective properties. For this reason, the anti-AChE activity of this family has been widely studied [15]. As can be seen in Table 1, *Lamiaceae* family extracts were generally stronger than the ones of the *Asteraceae* family. Out of the 42 ethanolic and aqueous extracts of 18 medicinal plants that were tested, 13 extracts showed high inhibitory activity, achieving values above 75%, whereas another 14 achieved moderate inhibition of the AChE (values between 50 and 75%) at a concentration of 250 μg/mL.

The ethanolic extract of the inflorescence, stem and leaf from *Lavandula latifolia* showed values higher than 90% at 125 μg/mL. The aqueous extract also showed similar values at 250 μg/mL. There is bibliographic information about the AChE inhibitory activity of the essential oils from *L. angustifolia and L. intermedia* [16], *L. luisieri* [17], *L. pedunculata* [18]*, L. stoechas* [19] and *L. viridis* [20]. However, no investigations about *L. latifolia* have been found.

In this study, differences were detected between four *Mentha* species, and the best inhibitory results were obtained with ethanolic and aqueous extracts from *M. longifolia*, at 77.98% and 90.45%, respectively. *M. aquatica*, *M. pullegium* and *M. suaveolens* showed lower activity. It is worth mentioning that there are similar data in the literature for the essential oil of the following aforementioned *Mentha* species: *M. longifolia* [21], *M. aquatic* [22], *M. arvensis* [23], *M. gentilis* [24], *M. piperita* and *M. spicata* [25], *M. pulegium* [26] and *M. suaveolens* [27].

The ethanolic extracts of aerial parts from two *Origanum vulgare* subspecies, *virens* and *vulgare*, showed similar results, with inhibition percentages of 91.75 ± 1.38 and 95.61 ± 2.02%, respectively. Both extracts also showed the lowest IC50 values, 4.62 ± 0.01 and 2.59 ± 0.01 μg/mL, which are even lower than values for galantamine (19.90 ± 4.80 μg/mL). These results are similar to those published by other authors for *O. vulgare* subspecies [28] and other closely related species such as *O. majorana* [29], *O. compactum* [30], *O*. *syriacum* [31] and *O. ehrenbergii* [32]. The difference between these works and our study relies on the polarity of the extracts. Most of the previous analyses were determined in essential oil or hydrophobic extracts (dichloromethane or ethyl acetate solvent).

A clear difference between the two extracts of *Prunella vulgaris* was found; whilst the ethanolic extract provided an inhibition percentage of 78.86 ± 7.39% at 250 μg/mL, the aqueous extract could be considered inactive (< 10%). To the best of our knowledge, there are only two literature data concerning the AChE inhibitory properties of the genus *Prunella*. Park et al. [33] studied the effects of the ethanolic extract of the flower of *P. vulgaris* var. *lilacina* on drug-induced memory impairment, concluding that this plant would be useful for treating cognitive impairments induced by cholinergic dysfunction and that it exerts its effects via NMDA receptor signaling. Qu et al. [34] determined that ethyl acetate extracts of *P. vulgaris* attenuated scopolamine-induced memory impairment in rats by improving behavioural performance and decreasing brain cell damage, which was associated with a notable reduction in AChE activity and MDA level, as well as an increase in SOD and GPx activities.

The following two different species of *Sideritis* have been analyzed: *S. hirsuta* and *S. hyssopifolia*. Only the aqueous extract of *S. hirsuta* showed high AChE inhibitory activity (93.27 ± 3.85%). No results about these species have been published; however, bibliographic information about the anti-AChE activity of this genus has been found for *S.* *arborescens* [35], *S. congesta* [36], *S. arguta, S. libanotica, S. perfoliata* and *S. pisidica* [37].

Ethanolic and aqueous extracts of *Teucrium chamaedrys* showed higher activity (98.09 ± 1.10% and 98.96 ± 9.85%) than galantamine at 250 μg/mL. These results are corroborated by the investigation of different species of *Teucrium* genus against AD, *T. arduini*, *T. chamaedrys, T. montanum* and *T. polium* [15], *T. hyrcanicum* [38] and *T. royleanum* [39]. The methanolic extract of *T. royleanum* and its fractions were also examined as inhibitors of acetylcholinesterase and a significant enzyme inhibition activity (52–83%) was found [39].

The genus *Thymus* contains about 350 species of aromatic perennial herbaceous plants and subshrubs. Many studies focused on the in vitro inhibitory activity of essential oil from the plants of this genus on acetylcholinesterase [40,41]. In this sense, our results are in accordance with them, ethanolic extract of *T. praecox* and *T. vulgaris* showed high AChE inhibition, 97.81 ± 10.90 and 82.48 ± 9.05%, respectively.

Eighteen extracts from seven different families (*Crassulaceae*, *Equisetaceae*, *Euphorbiaceae*, *Lytraceae*, *Papaveraceae*, *Primulaceae* and *Verbenaceae*) were studied. Six of them showed high AChE inhibition (>75%). The ethanolic extracts of *E. arvense* and *E. telmateia* demonstrated a similar effect and were more effective than the aqueous ones, with inhibitory values higher than 84% at 250 μg/mL. Since both species are close botanically and chemically, similar pharmacological results were to be expected.

*Euphorbiaceae* is a large family of flowering plants with around 300 genera and 7500 species. *Euphorbia* species contain glucosinolates and cyanogenic glycosides, such as linamarin, in different proportions. The quantitative difference in the chemical composition could justify the variability of results found between the aqueous extracts of the two species, *E. characias* (44.25 ± 5.01 mg/mL) and *E. helioscopia* (78.74 ± 4.15 mg/mL). Finally, the aqueous extracts of *Lytrum salicaria* (*Lytraceae*) and *Anagallis arvensis* (*Primulaceae*) and the ethanolic extract of *Papaver rhoeas* (*Papaveraceae*) also showed high AChE inhibitory activity (98.50 ± 13.50, 80.02 ± 1.25 and 99.78 ± 7.57 mg/mL, respectively). It is important to highlight the different chemical compositions of these species, *L. salicaria* is rich in tannins; *A. arvensis* in saponins and *P. rhoeas* in alkaloids. To the best of our knowledge, there is no anti-AChE activity reported in any of them, except for *Euphorbia* species, *E. antisyphlitica* [42], *E. characias* [43], *E. hirta* [44], *E. royleana* [45], *E. splendens* [46], *E. tirucalli* [47], *E. fischeriana* [48] and *Papaveraceae* [49].

**Figure 1:**
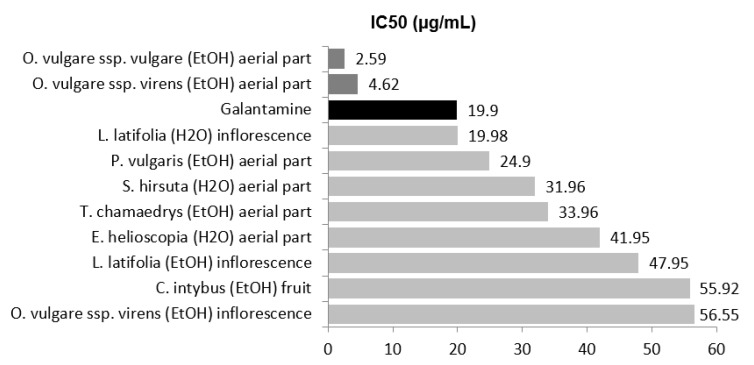
TOP 10 ranking of antioxidant extracts expressed as IC50 (µg/mL) values.

Half-maximal inhibitory concentration (IC50) is the most widely used measure of a drug’s efficacy in pharmacological research. It indicates how much drug is needed to inhibit a biological process down to half, thus providing a measure of the potency of an antagonist drug. The potential anti-AChE can be classified into the following categories based on the IC50 values: high potency, IC50 < 15 µM; moderate potency, 15 < IC50 < 50 µM; low potency, 50 < IC50 < 1000 µM [7]. Figure 1 shows the TOP 10 extracts in relation to their IC50 value and the comparison with galantamine.

Two alcoholic extracts presented higher anti-AChE potency than galantamine (19.9 ± 4.80 µg/mL), aerial parts of *O. vulgare* ssp. *virens* (4.62 ± 0.01 µg/mL) and *O. vulgare* ssp. *vulgare* (2.59 ± 0.01 µg/mL). The aqueous extract of inflorescence from *L. latifolia* showed an IC50 value (19.98 ± 0.49 µg/mL) equal to galantamine.

Based on the results of the screening, the ethanolic extract from aerial parts of *O. vulgare* ssp. *vulgare* showed the best anti-AChE activity, 7,7 times higher than galantamine. For this reason, the investigation continued with the chemical characterization of this extract. Antioxidant activity and total phenolic compounds were also determined. Finally, in order to establish structure-activity relationships, the results were analyzed by a correlation matrix.

### 2.2. Chemical Characterization of Origanum vulgare ssp. vulgare Aerial Parts

For chemical characterization, thin-layer chromatography, high-performance liquid chromatography with diode array detection (HPLC-DAD) and liquid chromatography-mass spectrometry (LC-MS) were used. Besides, the chemical characterization was complemented with the determination of total phenolic compounds.

#### 2.2.1. Total Phenolic Compounds Determination

Total phenolic compounds (TPC) were spectrophotometrically quantified following the Folin–Ciocalteu colorimetric method [50]. In this assay, phenolic compounds are oxidized in an alkaline medium by the Folin–Ciocalteu reagent (composed of a mixture of phosphowolframic acid and phosphomolybdenic acid) producing a reduced mixture of blue oxides of tungsten and molybdenum that can be quantified at 765 nm. The TPC of ethanolic extract was 207.64 ± 0.69 µg/mg of lyophilized extract. Previous studies with oregano also determined the TPC of the extracts [51], and sometimes they showed different results to the ones obtained in this work. To explain these differences, it is important to highlight that the chemical composition of an extract varies depending on the plant material, the growing conditions and the preparation method (solvent, time, temperature).

#### 2.2.2. Identification and Quantification of Main Groups of Phenolic Compounds by TLC and HPLC-DAD

**Figure 2:**
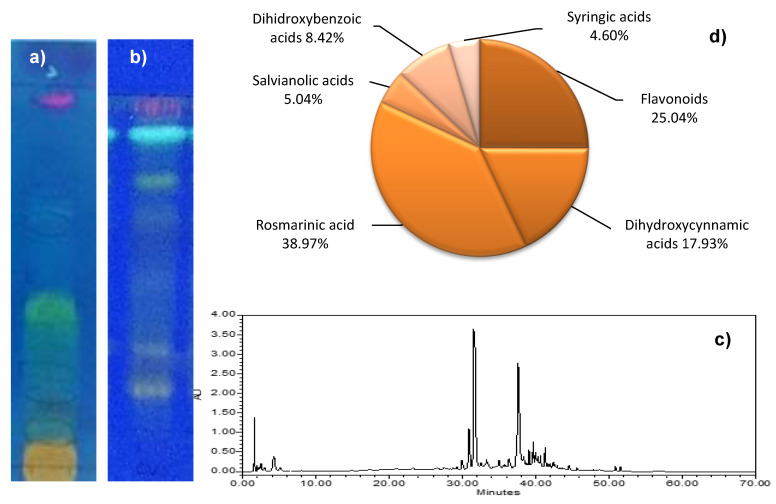
Chemical characterization of ethanolic extract from *Origanum vulgare* ssp. *vulgare*. (**a**) TLC plate with ethyl acetate:methanol:water (65:15:5, *v/v/v*) as mobile phase, and NP reagent; (**b**) TLC plate with ethyl acetate:glacial acetic acid:formic acid:water (100:11:11:26, *v/v/v/v*) as mobile phase, and NP reagent; (**c**) HPLC profile at 325 nm; (**d**) Quantification main groups of compounds expressed as percentage (%).

The chemical composition of the ethanolic extract of *O. vulgare* was firstly qualitatively analyzed by TLC with two different mobile phases (Figure 2a,b). Both TLC plates, after exposition to natural products reagent (NP), allowed the identification by the colour of the main compounds.

At the top of the TLC plate (Rf = 0.90) developed with ethyl acetate:methanol:water (65:15:5, *v/v/v*), a pink colored spot was detected. These spots could be chlorophylls since the aerial parts of *O. vulgare* were used as starting material. Wagner and Bladt [52] found similar fluorescent spots with high Rf values (> 0.70) on the TLC plate and identified them as chlorophylls. Chlorophylls are green pigments involved in photosynthesis and located in the leaves of plants. Blue spots, a characteristic colour of phenolic acids, with Rf = 0.70, 0.55, 0.40 and 0.25 were also detected. Phenolic acids have been described for *O. vulgare* [53,54,55], being the most importants 3,4-dihydroxybenzoic acid [56,57,58], rosmarinic acid [50,53,59] and caffeic acid [53,60,61]. TLC showed a yellow spot at the bottom of the plate (Rf = 0). This colour indicates the presence of flavonoids [52], potentially bioactive compounds already described in *O. vulgare* [50,55,62,63,64].

To confirm the chemical profile of the extracts, complementary TLC plates were prepared by modifying the mobile phase. According to Wagner and Bladt [52], ethyl acetate:glacial acetic acid:formic acid:water (100:11:11:26, *v/v/v/v*) is one of the best mobile phases to detect flavonoids and phenolic acids after NP treatment. At first sight, the separation of compounds was better than with the first mobile phase. The yellow spots at the baseline on the previous TLC were here separated into several spots. The presence of phenolic acids (in blue), flavonoids (in yellow), and chlorophylls (in pink) was also confirmed. The same profile was described previously in oregano hydroalcoholic extract [65].

TLC is a qualitative chromatographic technique in which neither the intensity of the bands should be used as a formal quantification nor the color given under certain conditions (reagent and observation wavelength) can be used for the identification of compounds beyond their chemical group (chlorophylls, flavonoids, phenolic acids...). To obtain quantitative results, techniques such as high-performance liquid chromatography (HPLC) should be used. The HPLC-DAD provides separation and the UV spectrum of compounds, allowing their assignment to a specific chemical group [66]. In this way, the peaks were grouped into the following six groups based on their UV spectrum: dihydroxycinnamic acids (λ_max_ 325–329 nm), dihydroxybenzoic acids (λ_max_ 220, 259.4, 293.7 nm), syringic acids (λ_max_ 220 sh, 260–280 nm), essential oils with an aromatic ring (λ_max_ 254 nm), salvianolic acids λ_max_ 289, 323 sh nm) and flavonoids (λ_max_ 254.6–267, 338–348.5 nm) (Figure 2c). The main compounds were luteolin derivative (31.4 min), 3,4-dihydroxybenzoic acid (31.8 min) and rosmarinic acid (37.9 min), previously described [65]. Dihydroxybenzoic acids, syringic acids, dihydroxycinnamic acids and salvianolic acids are phenolic acids obtained through the shikimic acid pathway in plants, but they were considered as different groups in the quantification and discussion of our results.

The area under curve (AUC) of each peak was transformed into concentration by linear regression analysis [65]. Ethanolic extract showed 15.56 ± 0.14 mg/100 mg of flavonoids expressed in terms of luteolin (#L9283, Sigma-Aldrich Co., St. Louis, MO, USA); 35.35 ± 1.13 mg/100 mg of dihydroxycinnamic acids (24,21 ± 1.08 mg was rosmarinic acid) and 11.14 ± 0.15 mg/100 mg of salvianolic acids expressed in terms of caffeic acid (#C0625, Sigma-Aldrich Co., St. Louis, MO, USA); 5.23 ± 0.04 mg/100 mg of dihydroxybenzoic acids and 2.89 ± 0.24 mg/100 mg of syringic acids expressed in terms of 3,4-dihydroxibenzoic acid (#D109800, Sigma-Aldrich Co., St. Louis, MO, USA). Essential oils typical of oregano showed low-intensity peaks in HPLC-DAD, so they were not considered in the chemical quantification. Figure 2d shows the distribution of the main group expressed in percentage. The most abundant compounds are dihydroxycinnamic acids (56.90%), with rosmarinic acid being the highest percentage (38.97%) and flavonoids (25.94%).

#### 2.2.3. Identification of Main Compounds by LC-ESI-QTOF-MS

After separating compounds from a sample by liquid chromatography (HPLC-DAD), highly sensitive instrumental analytical techniques, such as mass spectrometry (LC-ESI-QTOF-MS), can be applied for the identification of individual compounds. This technology is based on the ionization of the separated compounds to obtain structural information [67]. A large number of secondary metabolites are glycosylated compounds and the fragmentation by LC-MS allows the revealing of the main structure and the attached sugars, making it a useful technique for the phytochemical identification of compounds extracted from plants.

The peaks were preliminarily assigned to a family of phenolic compounds based on their UV-vis spectra. The structure of each compound was proposed based on fragmentation patterns using ESI-MS-MS experiments as well as by co-elution with several standards. In total, 23 compounds were thus detected and identified or tentatively identified. They are listed in Table 2, with UV-visible and MS data.

**Figure 3:**
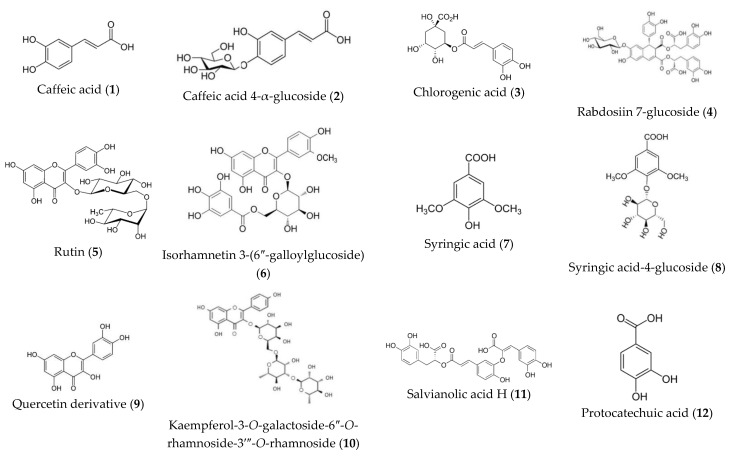

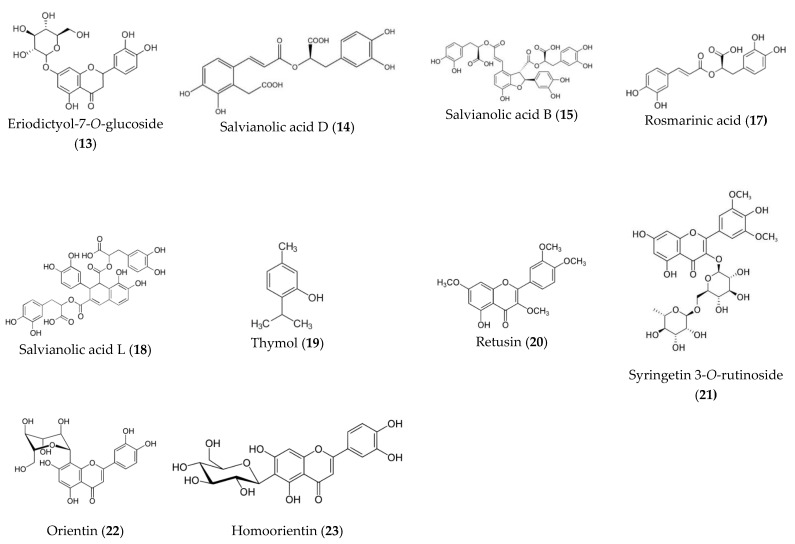
Main compounds of ethanolic extract from *Origanum vulgare* ssp*. vulgare.* Dihydroxycinnamic acids (**1–4**, **17**); dihydroxybenzoic acids (**12**); syringic acids (**7**, **8**); essential oil (**19**); salvianolic acids (**11**, **14**, **15**, **18**); flavonoids (**5**, **6**, **9**, **10**, **13**, **21**–**23**).

Figure 3 shows the structures of identified compounds. The identification of tentatively characterized compounds present in the oregano aerial part’s extract is explained below.

Dihydroxycinnamic acids (λ_max_ 316–331 nm) were detected first in LC-MS analysis. They were also previously described in *Lamiaceae* species [53,58,68]. These compounds could be related to the blue spots on TLC (mobile phase: ethyl acetate:glacial acetic acid:formic acid:water (100:11:11:26, *v/v/v/v*)).

One monomer, caffeic acid (compound **1**), was identified at 0.9 min (*m/z* 179.05, λ_max_ 296sh, 324 nm). This compound yielded an ion at *m/z* 179.05 [M-H]^−^ and a prominent fragment at *m/z* 135.04 [M-H-44]^−^ through the loss of a CO_2_ group. The extract also showed four more complex forms of caffeic acid (compounds **2**, **3**, **4** and **17**). Fragment *m/z* 179.05 of caffeic acid appears in the mass spectra of all of them. Compound **2** at 1.1 min showing [M-H]^−^ at *m/z* 341.07 and [M-H-162 (glucose residue)]^−^ at *m/z* 179.03 was tentatively characterized as caffeic acid 4-α-D-glucoside. The loss of 162 amu is likely due to the cleavage of a glucose moiety. Chlorogenic acid (compound **3**), a combination of caffeic acid and quinic acid (*m/z* 354.31), was also detected at 1.3 min. The highly intense characteristic ion at *m/z* 191.01 [M-H-162 (caffeoyl residue)]^−^ corresponding to quinic acid confirmed the structure. Compound **17** was assigned to rosmarinic acid, an ester of caffeic acid and 3,4-dihydroxyphenyl lactic acid and it is widely described in *Lamiaceae* and *Boraginaceae* families. The MS fragmentation of rosmarinic acid pseudomolecular ion (*m/z* 358.97) lead to three peaks at *m/z* 197.01 [M-H-162 (caffeoyl)]^−^, 179.05 [M-H-180] and 161.02 [M-H-198]^−^, corresponding to the deprotonated form of 3-(3,4-dihydroxyphenyl) lactic and caffeic acids and their dehydrated forms. These results agree with the fragmentation scheme proposed by Lecomte et al. [69]. Rosmarinic acid, whose name derives from *Rosmarinus officinalis* L., has been identified as one of the most active compounds in several plants from the *Lamiaceae* family, such as rosemary and oregano [70]. Its identification by HPLC-DAD and LC-MS is widely reported in the literature [20,50,58,71]. Definitive elucidation of these structures was also confirmed by co-injection with reference standards. Compound **4** was identified as rabdosiin 7-*O*-β-D-glucoside (*m/z* 879.05 [M-H]^−^, λ_max_ 287 sh, 329 nm). This compound is a caffeic acid tetramer connected to a lignan skeleton (*m/z* 718.6) and a glucose unit [M-H-162]^−^. Originally, rabdosiin has been isolated and identified from the stem of *Rabdosia japonica* Hara, *Labiatae* [72]. According to published data, it has been suggested as a potential anti-HIV, antiallergic, and antiproliferative agent [73]. This is the first report about the presence of rabdosiin 7-*O*-β-D-glucoside in *Origanum* species.

The second group of compounds, dihydroxybenzoic acids from the shikimic acid pathway, was identified. These acids, showing a blue spot on the TLC plate, can be the result of the transformation of caffeic acid and have been described in previous studies with oregano [57,74]. According to the retention time (6.5 min), UV-spectra (λ_max_ 217.0, 261.7, 294.9 nm), *m/z* 153.01 [M-H]^−^ and a fragment ion [M-H-44 (CO_2_)]^−^ at *m/z* 109.02, compound **12**, the major component, could be identified as the 3,4-dihydroxibenzoic acid or protocatechuic acid, also previously described in *O. vulgare* [66,75,76]. Final identification was carried out by co-injection of 3,4-DHBA standard (#D109800, Sigma-Aldrich Co., St. Louis, MO, USA).

The third type of UV peak (λ_max_ 220 sh, 280 nm) corresponds to the group of syringic acids, already described in *O. vulgare* [54,63,77]. Syringic acids are phenolic compounds strictly named 4-hydroxy-3,5-dimethoxybenzoic acids, synthesized from ferulic acid and caffeic acid by a series of enzymatic reactions in the shikimic acid pathway [70,78]. Despite being derivates of dihydroxybenzoic acid, they present a different UV spectrum. For this reason, these compounds are treated separately. Two syringic acids were detected (compounds **7** and **8**), at 3.4 and 3.7 min. Compound **7** was identified as syringic acid (*m/z* 197.03 [M-H]^−^) and compound **8** as a glycosylated variation, syringic acid-4-β-D-glucoside (at *m/z* 359.08 [M-H]^−^ and *m/z* 197.04 [M-H-162]^−^.

Most of the published studies on oregano use essential oils as plant material due to the important bioactivity of these compounds [74,79,80]. As they are volatile, only one essential oil (compound **19**) was detected in this study. Compound **19**, (λ_max_ 254.4 nm, *m/z* 149.1 [M-H]^-^) is identified as thymol, the most important essential oil in oregano. Final identification was carried out by co-injection of the thymol standard.

The next group was one of the salvianolic acids, which was also previously reported in oregano [74,81,82]. From a chemical point of view, they are considered a large group of acids whose names are attributed with letters as follows: salvianolic acid A, B, E,… These compounds have a complex chemical structure derived from rosmarinic acid, and they were distinguished from dihydroxycinnamic acids because they showed a different UV spectrum (λ_max_ 289, 323 sh nm). In LC-MS, four salvianolic acids were identified (compounds **11**, **14**, **15**, **16** and **18**). Fragments *m/z* 179.04 of caffeic acid appear in the mass spectra of all detected salvianolic acids. Compound **11** (6.3 min), caffeic acid trimer with deprotonated ion [M-H]^−^ at *m/z* 537.09 was assigned as salvianolic acid H or salvianolic acid I (pair of isomers). Compounds **14** (6.8 min), **15** (7.1 min) and **18** (7.5 min), caffeic acid tetramers, generated the same pseudomolecular ion [M-H]^−^ at *m/z* 717.12 and were identified as salvianolic acid E, salvianolic acid B and salvianolic acid L, respectively. Finally, a caffeic acid hexamer, compound **16**, at 7.6 min and with a pseudomolecular ion *m/z* of 987.22 was detected. The final identification of these compounds was determined by comparison with retention times and MS fragmentation data [83], except for compound **16**, whose structure was not completely elucidated.

The last chemical group present in oregano and *Lamiaceae* is flavonoids [71,84]. These secondary metabolites are generally present in glycosylated forms, with the main molecule attached to one or more sugars (glucose, galactose) [85]. In UV-spectra, two separated and characteristic shoulders easily identify these compounds. Nine flavonoids with three different types of spectra were detected. Compounds **5**, **6**, **9**, **10** and **21** showed typical UV spectra of the flavonol. Compounds **5** (2.4 min) showed molecular ions at *m/z* 609.17 [M-H]^−^ and was identified as rutin (quercetin-3-*O*-rutinose, λ_max_ 254.6, 348.5 nm), producing an MS ion at *m/z* 463.3 [M-H-146]^−^, by loss of rhamnose moiety and a quercetin ion at *m/z* 301.8 [M-H-146-162]^−^. The loss of 308 amu is characteristic of compounds having rutinose. In a similar way, compound **21** (8 min) was identified as syringetin 3-*O*-rutinoside, *m/z* 653.14 [M-H]^−^, 507.4 [M-H-146]^−^ and 345.07 [M-H-146-162]^−^. Syringetin is a dimethoxyflavone, myricetin, in which the hydroxy groups at positions 3′ and 5′ have been replaced by methoxy groups. The ion *m/z* 330.1 [M-H-308-15]^−^ and 315.2 [M-H-308-30]^−^ confirmed the presence of two -OCH_3_ groups. Compound **10** (5.8 min) with λ_max_ 266, 346 nm in the DAD spectrum and showing a molecular anion at *m/z* 739.05 in the negative ESI spectra, and ions at *m/z* 659.07 [M-H-146]^−^, *m/z* 447.01, [M-H-146-146]^−^ was identified as a kaempferol-3-galactoside-6″-rhamnoside-3′″-rhamnoside. Cleavage of this glycoside gave the aglycone at *m/z* 285.03 [M-H]^−^, kaempferol. Compound **6** (2.5 min) was an isorhamnetin derivate with *m/z* 315.02 [M-H-314]^−^ corresponding to the aglycone that has lost galloylhexoside fragment. The mass spectrum of compound **6** showed the fragment *m/z* 447.03 [M-H-152]^−^ corroborating the galloyl substitution. Moreover, the additional mass loss of 162 amu confirmed the presence of a hexoside (glucoside or galactoside). Compound **6** was thus identified as isorhamnetin 3-(6″-galloylglucoside). Finally, a quercetin oxalate was detected at 5.6 min (compound **9**) with *m/z* 388.20 [M-H]^−^ and *m/z* 301.80 [M-H-87]^−^.

Compound **20** (7.9 min) is a 5-hydroxy-3,3′,4′,7-tetramethoxyflavone, namely, retusin with the ion *m/z* 357.09 [M-H]^−^ and λ_max_ 350, 268 nm. The ions at *m/z* 342.12 [M-H-15]^−^, 327.07 [M-H-15]^−^, 312.02 [M-H-15]^−^ and 297.02 [M-H-15]^−^ confirmed the four methoxyl groups.

In the MS identification of *C*-glycosides, the key fragmentations used were [M-60]^−^, [M-90]^−^, [M-120]^−^ and [M-240]^−^. Compounds **22** (orientin) and **23** (homoorientin) were identified as *C*-glycosyl derivatives of luteolin (λ_max_ 254, 267 nm). Ion fragmentation of both were *m/z* 447.05 [M-H]^−^, 357.78 [M-H-90]^−^ and 327.22 [M-H-120]^−^ and 285.5 [M-H-162]^−^.

Finally, one flavanone glycoside (compound **13**) at 6.6 min and λ_max_ 285, 325 nm was identified as eriodictyol-7-*O*-glucoside. Ions at *m/z* 449.19 [M-H]^−^ and *m/z* 287.05 [M-H-162]^−^ confirmed the structure.

Rosmarinic acid, apigenin, luteolin and quercetin are the most recurrent compounds in this *Lamiaceae* species [50,59,86]. With increasing evidence of the biological activity of flavonoids and phenolic acids from oregano species, quantification of these compounds is important. Reports from different oregano species have shown that flavones are the most abundant flavonoid subgroup, followed by flavonols, flavanones and flavanols [87]. The most common phenolic acids in oregano are hydroxycinnamic acid and hydroxybenzoic acid derivatives [87]. However, their content and distribution can vary depending on geographical, environmental growing factors and the vegetative stage of the plant [88,89], showing a different chemical profile within the same species [54]. For these reasons, it is very important for the chemical characterization and the establishment of quimiotaxonomic markers for each species and subspecies. In aerial parts of *O. vulgare* spp *vulgare*, rosmarinic acid and 3,4-dihydroxybenzoic acid could be two optimal candidates for markers.

### 2.3. Antioxidant Activity

Phenolic compounds are secondary metabolites present in a wide range of medicinal plants with a chemical structure that can act as an H donor, making them potentially antioxidant compounds. Molecular oxygen (O_2_) is involved in metabolic functions. However, it can also be present as short-lived highly reactive derivatives (reactive oxygen species—ROS) as the result of these enzymatic reactions. Superoxide (O_2_^•−^), hydrogen peroxide (H_2_O_2_) and the hydroxyl radical (^•^OH) are some of these derivatives that can cause cell damage [90]. They can affect DNA and polyunsaturated fatty acids in the membrane [91]. Organisms are prepared to counteract these damages through antioxidant defense systems. However, according to the aging and free radical theory, the effectiveness of these protective systems tends to decrease with age, and the accumulation of these harmful molecules can create pathologies in the body, developing diseases such as Alzheimer’s and diabetes [90,92]. Most of the current research with natural products is focused on finding external co-adjuvants to counteract this oxidative damage, either as prevention or treatment [93,94]. Compounds that are able to counteract this oxidative damage are called antioxidants. As an exogenous aid to prevent damage to the body, these antioxidant compounds can reduce the formation of these free radicals or neutralize them [90].

#### 2.3.1. Antioxidant Activity In Vitro against DPPH Radical

Among in vitro assays, the DPPH^•^-based method is probably the most popular one due to its simplicity, speed and low cost. DPPH^•^ (1,1-diphenyl-2-picrylhydrazyl) is a stable free radical that can be reduced by transferring hydrogen from other compounds. Since 1995, when Brand-Williams first published and discussed in depth the methodology [95], some variants have been developed. Depending on the equipment and the interest of the study, the reaction can be quantified at a pre-defined time (30 min. mainly) or kinetic studies can be performed. Nonetheless, the principle of the reaction is always the same as follows: the reduction of DPPH^•^ is followed by monitoring the decrease in its absorbance at a characteristic wavelength during the reaction. In its radical form, DPPH^•^ absorbs at 517 nm, but upon reduction by an antioxidant (AH) or a radical species (R^•^), the absorption disappears.

In fact, as Brand-Williams recommends, the reaction was monitored over time to establish a kinetic scale depending on the stabilization time-point of the reaction. A sample is considered a *fast* antioxidant if the stabilization point of the reaction is reached before 30 min, an *intermediate* antioxidant if it stabilizes between 30 and 60 min, and a *slow* antioxidant if it needs more than 60 min to stabilize. The reaction is stable when no statistical differences (*p* > 0.05) are observed between two consecutive values [96]. Ethanolic extract was an intermediate antioxidant (stabilization points between 30 and 60 min) with an IC50 = 3.22 ± 0.19 µg/mL at 60 min (Table 3).

IC50 values of antioxidant activity depend on the concentration of DPPH, and this makes difficult the comparison with other published studies. Nevertheless, the antioxidant activity index (AAI), which is independent of the concentration of DPPH, can be calculated by dividing the concentration of DPPH in the final solution (20 µg/mL) by the IC50 value [97]. This index determines the strength of the antioxidant activity regardless of the concentration of DPPH. According to the current classification, plant extracts are considered *poor* antioxidants when AAI < 0.5, *moderate* when AAI is between 0.5 and 1.0, *strong* if AAI is between 1.0 and 2.0 and *very strong* antioxidants when AAI > 2.0. In this sense, the antioxidant activity index (AAI) was also calculated to determine the strength of the antioxidant activity of the extracts regardless of the concentration of DPPH. The results showed that the extract was a *very strong antioxidant*, with an AAI = 6.21 ± 0.10 (Table 3).

#### 2.3.2. Antioxidant Activity In Vitro against ABTS Radical

As a complement to DPPH antioxidant determination, the Trolox equivalent antioxidant capacity (TEAC) method, also known as the ABTS radical cation decolorization assay, was performed in vitro. This assay determines, through a simple and inexpensive protocol, the ability of an antioxidant compound to counteract the free radical ABTS. Unlike other common in vitro antioxidant tests to determine this activity, this method does not require enzymes or special conditions [94]. In addition, the method could be applicable to the study of hydrophobic and hydrophilic antioxidants. The ABTS in vitro assay was carried out according to García-Herreros et al. [98]. It is based on the formation of an ABTS cation radical that exhibits a colour change that is measurable by spectrophotometry at 741 nm. The assay was performed with the extract at a 1 mg/mL concentration and the results were expressed as the amount of Trolox (TE) per mg of lyophilized extract, after substituting the data in the Trolox calibration curve. The antioxidant activity was 34.24 ± 0.20 mg/100 mg of extract.

### 2.4. Chemical Composition—Biological Activity Relationship

Correlation is a type of association between two countable variables that evaluates the trend in the data (positive or negative). In a correlation, a positive value indicates a positive direct relationship, while a negative value indicates a negative indirect relation between the variables. The magnitude indicated the strength of the link, being values between −1 and 1. The closer to the unit, the stronger the relationship, which on a graph is generally observed as a smaller dispersion of the values. One of the most widely used coefficients for calculating lineal correlation is Pearson’s, which assumes that the trend must be linear, there are no outliers and the variables must be numeric with a reasonable number of values.

The Pearson correlation coefficients, which show the relationship between the biological activity of the ethanolic extract of *O. vulgare* ssp. *vulgare* and its chemical composition, are presented in Table 4.

The AChE-IC50 activity was strongly correlated (R^2^ > 0.85) in a linear, negative manner to antioxidant activity (DPPH-AAI (R^2^ = −0.8649) and ABTS (R^2^ = −0.9487)), syringic acids (R^2^ = −0.9864), flavonoid (R^2^ = −0.9563) and dihydroxybenzoic acids’ (R^2^ = −0.9247) content. A moderate and negative correlation (R^2^ = 0.76) between AChE-IC50 activity and dihydroxycinnamic and rosmarinic acid content was also observed. Free DPPH radical scavenging activity, expressed as antioxidant activity index (AAI), had a strong correlation to ABTS activity (R^2^ = 0.9378) and all the main compounds analyzed (R^2^ > 0.9). These results are according to many studies of the activity of polyphenols as AChE inhibitors, which, in addition to inhibiting AChE activity, also have an antioxidant effect, including scavenging free radical forms of oxygen and the ability to chelate transition metals, which reduces the formation of inflammation that can cause the destruction of neuronal structures [99].

The neuroprotective effect of flavonoids and dihydroxycinnamic acids has been widely studied by many authors [2,15,100]. The inhibitory effect on AChE activity was also reported for individual phenolic acids, in the following order: rosmarinic acid > caffeic acid > gallic acid = chlorogenic acid > homovanillic acid > sinapic acid. Flavonoids, such as quercetin, kaempferol and, to a lesser extent, luteolin were also reported as efficient AChE inhibitors [101].

However, it is important to highlight the AchE activity of syringic acids. To our knowledge, there are currently a few works focused on them [102]. Syringic acids show a wide range of therapeutic applications in the prevention of diabetes, CVDs, cancer, cerebral ischemia; antioxidant, antimicrobial, anti-inflammatory, antiendotoxic, neuro- and hepatoprotective activities have been described [103]. Recently, a study analyzed 16 hydroxybenzoic acids using calorimetry and docking simulation as AchE inhibitors. All tested compounds were shown to inhibit the hydrolysis of ACh, and the best properties were shown by methyl syringinate; syringic acid also showed a high inhibition percentage [104]. Considering that AChE inhibitory potential has been mainly investigated for essential oils in the *Lamiaceae* family, these findings suggest the great influence of other chemical constituents such as syringic, which may have great relevance in pharmacological fields and open a new research line.

## 3. Materials and Methods

### 3.1. Plant materials and extraction

Plants were collected in Santacara, Navarra, Spain, (Longitude: O1°32′38.33″ and Latitude: N42°22′47.71″) and identified by the botanist, Dr. Rita Yolanda Cavero. Voucher specimens have been deposited in the PAMP Herbarium of the University of Navarra. Plants were air-dried in the dark at room temperature. All species are listed in Table 1.

Plant materials (10 g) were ground into fine powder (180 mesh) and extracted by maceration with 250 mL of ethanol (EtOH) and water (H_2_O) at room temperature in a closed container (3 times each 24 h). The extracts were dried under reduced pressure at 30 °C in a rotary evaporator (Buchi R-300) and then were lyophilized (Virtis BT3-SL, NY, EEUU). Finally, the dry extracts were stored in glass vial at −80 °C.

### 3.2. Antiacetylcholinesterase Activity

A qualitative antiacetylcholinesterase activity was studied by TLC according to the method described by Uriarte-Pueyo and Calvo [105]. Extracts and galantamine (#Y0001191, Sigma-Aldrich Co., St. Louis, MO, USA) were spotted at 0.20 mg onto the TLC plate and developed with ethyl acetate:methanol:water (65:15:5, *v/v/v*) as mobile phase. Then, the plates were sprayed with DTNB or 5,5′-dithiobis(2-nitrobenzoic acid) (#D218200, Sigma-Aldrich Co., St. Louis, MO, USA)/ATCI or acetylthiocholine iodide (#01480, Sigma-Aldrich Co., St. Louis, MO, USA) 1:1. It was allowed to dry for 3–5 min and 3 U/mL of acetylcholinesterase (AchE) (#C2888, Sigma-Aldrich Co., St. Louis, MO, USA) solution was sprayed. After AChE application, a yellow background appeared, with white spots for AChE inhibiting extracts or compounds.

Quantitative AChE inhibitory activity was measured by spectrophotometric method developed by Rhee et al. [106] and modified by Carpinella et al. [107]. The lyophilized extracts were diluted in their corresponding solvent (ethanol or water) to give a stock solution of 20 mg/mL and three serial solutions were prepared (10–2.5 mg/mL). Twenty-five μL of each solution was added to 25 μL of 15 mM ATCI, 125 μL of 3 mM DTNB, 25 μL of acetylcholinesterase and 5.0 μL of 0.1 mM sodium phosphate buffer (pH 8.0) into a 96-well microplate and incubated for 15 min at 25 °C. The hydrolysis of acetylthiocholine iodide was monitored by the formation of the yellow 5-thio-2-nitrobenzoate anion as a result of the reaction of DTNB with thiocholine, catalyzed by enzymes. Absorbance was read at a wavelength of 405 nm using a PowerWave™ Microplate Spectrophotometer (BioTek, Winooski, VT, EEUU) and results were processed with KC Junior BioTek data analysis software. Inhibition (%) of AChE was calculated by using the following equation: Inhibition (%) = [1-(*A_samp_*/*A_con_*)/*A_std_*] × 100, where *A_samp_*, *A_con_* and *A_std_* are the absorbances measured with a sample, with sample but without enzyme and without a sample, respectively. The inhibitory concentration (IC50) was calculated by GraphPad Prism v 4.00 analysis. Galantamine, dissolved in methanol, was used as a positive control. Each measurement was made at least in triplicate.

### 3.3. Chemical Characterizationof Origanum vulgare ssp. vulgare Aerial Parts

#### 3.3.1. Total Phenolic Compounds Determination

Total phenolic compounds (TPC) were spectrophotometrically quantified following the Folin–Ciocalteu colorimetric method [50]. The ethanolic extract of *O. vulgare* ssp. *vulgare* was dissolved in ethanol at 1 mg/mL. For the reaction, 15 µL of sample were mixed with 75 µL of Folin–Ciocalteu reagent (#47641, Sigma-Aldrich Co., St. Louis, MO, USA) allowing to react for 2 min. Ethanol was used as a blank sample. Then, 225 µL of Na_2_CO_3_ and 1,185 µL of distilled water were added and, after shaking, the mixture was incubated at room temperature for 2 h.

In a 96-well plate, 300 µL of the solution was disposed per well and the absorbance at 765 nm was monitored. The absorbance was transformed into µg of gallic acid per mg of lyophilized extract by extrapolation from a previously obtained calibration curve (*y* = 0.001*x* + 0.0038, R^2^ = 0.999, where *y* corresponds to absorbance and *x* to gallic acid concentration).

#### 3.3.2. Identification and Quantification of Main Groups of Phenolic Compounds by TLC and HPLC-DAD

This activity was firstly confirmed by using thin-layer chromatography (TLC) as a qualitative assay disposing 10 μL of hydroalcoholic extract (10 mg/mL) in a Silicagel 60 F_254nm_ with plastic base (#105554, Merck KGaA, Darmstadt, Germany) that were eluted with ethyl acetate:methanol:water (65:15:5, *v/v/v*) and ethyl acetate:acetic acid:formic acid:water (100:11:11:26, *v/v/v*) in a chromatography chamber. Spots were observed at 366 nm after treatment with NP reagent (#126705, Sigma-Aldrich Co., St. Louis, MO, USA).

Then, the main groups of compounds of the extract were qualitative and quantitatively identified by high-performance liquid chromatography with diode array detector (Waters HPLC 600E multi-solvent delivery system, a Waters U6K sampler and a Waters 991 photodiode-array detector, Waters Corp., Milford, MA, USA). Samples were injected in a C18 reversed-phase column (Nova-Pak 15 0 mm × 3.9 mm, 4 μm, Waters Corp., Milford, MA, USA) at 25 °C with a flow rate of 0.8 mL/min and were eluted with acetonitrile (solution A) and acidified water type I adjusted to pH 2 with formic acid (solution B), in different proportions (%) of solution B: 0–10 min, 95%; 10–20 min, 95–90%; 20–35 min, 90–80%; 35–45 min, 80–60%; 45–50 min, 80–20% and then 95% in 5 min. The range of detection was established between 190 and 600 nm. Quantification of the main groups of compounds was carried out according to the previously published method by our group [65]. The areas under the curve (AUC) of the main peaks were expressed in terms of mg of the standard compound per 100 mg of extract by linear regression analysis.

#### 3.3.3. Identification of Main Compounds by LC-ESI-QTOF-MS

The individual compounds were identified by LC-ESI-QTOF-MS (Ultimate 3000 RSLCnano system (Thermo Fischer Scientific, Idstein, Germany) interfaced with a quadrupole time-of-flight (QqToF) Impact II mass spectrometer equipped with an electrospray source (Bruker Daltonics, Bremen, Germany) [108]. Conditions of the method applied were the following: column Nova-Pack^®^ C18 (150 × 2.1 mm, 1.7 µm) as Stationary phase, at 25 °C with a flow rate of 0.8 mL/min and were eluted with distilled water (0.1% formic acid) (solution A) and acetonitrile (0.1% formic acid) (solution B) as mobile phase, in different proportions (%) of solution B: 0–1.5 min, 5%; 1.5–13 min, 5–75%; 13–18 min, 75–100%; 18–21 min, 100%; 21–23 min, 100–50% and then 5% in 7 min. Optimized parameters were set as ion spray voltage, +4.5/−2.5 kV; end plate offset, 500 V, nebulizer gas (N_2_), 2.8 bars; dry gas (N_2_), 8 L/min; dry heater, 200 °C. Internal calibration was performed in High-Precision Calibration (HPC) mode with a solution of sodium formate 10 mM introduced into the ion source via a 20 µL loop at the beginning of each analysis using a six-port valve. Acquisition was performed in full-scan mode in the *m/z* 50–1300 range, and in a data-depending MS/MS mode with 3 Hz acquisition using a dynamic method with a fixed cycle time of 3 s. The duration of dynamic exclusion was 0.4 min. The acquired data were processed by Data Analysis 4.1 software (Bruker Daltoniks, Bremen, Germany). The peaks were automatically numbered and the mass of the fragmentation was compared with the data obtained from the PubChem online database.

### 3.4. Antioxidant Activity

#### 3.4.1. Antioxidant Activity In Vitro against DPPH Radical

Antioxidant activity can be monitored using the scavenging effect of radicals on DPPH^•^ (#D9132, Sigma-Aldrich Co., St. Louis, MO, USA), which changes from purple to yellow in the presence of an antioxidant compound. This change can be quantified by spectrophotometry at 517 nm (spectrophotometer UV PowerWave XS, BioTek Instruments, Inc., Winooski, VT, USA) according to the method previously described [95]. The results were expressed as scavenging activity (percentage of inhibition, %) and IC50, the concentration in which the 50% of the free radical DPPH^•^ is reduced. Furthermore, by using IC50 values the index of antioxidant activity (AAI) was calculated with the following formula: AAI = final DPPH concentration (µg/mL)/IC50 (µg/mL).

#### 3.4.2. Antioxidant Activity In Vitro against ABTS Radical

The ABTS (#10102946001, Sigma-Aldrich Co., St. Louis, MO, USA) in vitro assay was carried out according to García-Herreros et al. [98]. The absorbance at 741 nm was measured with an FLUO Star Omega spectrofluorometric analyser (BMG Labtechnologies, Offenburg, Germany). The results were expressed in terms of Trolox (6-hydroxy-2,5,7,8-tetramethylchroman-2-carboxylic acid, TE). Data transformation was obtained by extrapolation from the Trolox calibration curve whose equation was *y* = 0.2802*x* + 0.8694, R^2^ = 0.9952, where *y* is the inhibition percentage (% *I*) and *x* corresponds to Trolox concentration (mM).

### 3.5. Statistical Analysis

Means, standard deviations and graphs were obtained with Microsoft Excel 2013 (Microsoft Corp., Redmond, WA, USA). The experiments were performed in triplicate. Statistical analysis was performed using Stata v.12 (StataCorp LLC, College Station, TX, USA) and differences were calculated on each pair of interest by two-tailed, equal variance Student *t*-test. They were considered significant at *p* < 0.05. The relationship between TPC, individual groups of compounds and the antioxidant and AChE inhibition activity was analyzed by Pearson correlation coefficients.

## 4. Conclusions

The alcoholic and aqueous extracts of plants used in the traditional medicine of Navarra for neurological diseases were screened for AChE inhibition. The inhibitory activities of these extracts support the traditional use of these species. In total, 21 out of 90 extracts showed a high AChE activity (75–100 % inhibition). Among them, the ethanolic extract from aerial parts of *Origanum vulgare* ssp. *vulgare* was selected as a promising candidate for a source of potent AChE inhibitor as well as an antioxidant agent. A phytochemical investigation of the extract resulted in 23 phenolic compounds. Among these, syringic acids could be interesting due to their neuroprotective and antioxidant effects. Further evaluation is required to assess their safety and bioavailability in vivo animal models.

Considering that *O. vulgare* L. comprises several subspecies such as *hirtum* (Link) Ietsw., *vulgare* L., *viridulum* (Martrin-Donos) Nyman, *glandulosum* (Desfontaines) Ietswaart, gracile (Koch) Ietsw., *virens* (Hoffmanns. & Link) Ietsw., and *viride* L., further studies of these subspecies should be carried out in order to look for leads for the treatment of Alzheimer and other neurological diseases.

## Figures and Tables

**Table 1:** Yields (*w/w* %) and acetylcholinesterase inhibitory activity, expressed as percentage (%) and IC50 (μg/mL) of the ethanolic and aqueous extracts.

		Ethanolic Extract					Aqueous Extract				
		Yield (*w/w* %)	Inhibitory Activity (%)			IC50 (μg/mL)	Yield (*w/w* %)	Inhibitory Activity (%)			IC50 (μg/mL)
			250 μg/mL	125 μg/mL	62.5 μg/mL			250 μg/mL	125 μg/mL	62.5 μg/mL	
***Asteraceae***											
*Achillea millefolium* L. ssp. *millefolium*	Leaf	8.39	56.96 ± 4.93	45.17 ± 6.10	38.70 ± 6.43	190.80 ± 8.02	10.7	<10	n.q.	n.q.	n.d.
	Stem	8.47	<10	n.q	n.q.	n.d.	4.66	46.89 ± 4.29	32.57 ± 5.08	21.99 ± 5.83	>250
	Inflorescence	11.99	60.78 ± 5.21	49.40 ± 6.14	39.00 ± 2.51	128.87 ± 2.57	9.73	38.95 ± 5.93	35.40 ± 6.03	32.16 ± 3.96	>250
*Anthemis arvensis* L. ssp. *arvensis*	Aerial part	20.09	53.80 ± 5.01	33.82 ± 6.80	29.24 ± 1.93	234.76 ± 5.54	10.26	67.17 ± 15.30	46.53 ± 4.79	36.73 ± 2.12	158.84 ± 1.58
*Anthemis cotula* L.	Aerial part	13.83	56.50 ± 5.96	49.62 ± 5.04	38.43 ± 5.61	156.84 ± 3.76	10.87	<10	n.q.	n.q.	n.d.
*Cichorium intybus* L.	Fruit	9.68	66.57 ± 9.31	63.49 ± 1.06	61.24 ± 1.19	55.92 ± 4.75	14.82	60.08 ± 3.06	35.22 ± 4.79	30.82 ± 2.51	189.81 ± 2.57
*Helichrysum stoechas*	Aerial part	4.42	<10	n.q.	n.q.	n.d.	8.0	49.57 ± 7.64	29.48 ± 4.48	25.52 ± 4.92	>250
*Jasonia glutinosa*	Inflorescence	5.68	57.81 ± 6.99	48.60 ± 8.30	33.02 ± 7.15	239.76 ± 13.17	13.53	56.82 ± 1.90	56.10 ± 7.69	52.93 ± 4.55	60.83 ± 9.80
*Jasonia tuberosa*	Aerial part	17.17	<10	n.q.	n.q.	n.d.	15.79	55.94 ± 4.25	30.35 ± 1.01	28.67 ± 5.63	219.10 ± 5.73
*Santolina chamaecyparesus* L. ssp. *squarrosa* Nyman	Inflorescence	13.15	75.82 ± 6.69	52.18 ± 6.00	24.20 ± 6.57	100.90 ± 5.74	8.43	<10	n.q.	n.q.	n.d.
*Sylibum marianum* (L.) Gaertner	Inflorescence	5.39	49.79 ± 2.44	22.43 ± 8.35	16.45 ± 0.42	>250	6.87	<10	n.q.	n.q.	n.d.
*Tanacetum parthenium* (L.) Schultz	Stem	10.25	40.83 ± 6.77	39.69 ± 5.02	14.71 ± 2.14	>300	8.21	53.46 ± 5.22	38.86 ± 4.02	40.96 ± 8.15	193.80 ± 4.25
	Leaf	15.61	67.57 ± 1.25	59.66 ± 19.73	48.53 ± 2.24	92.80 ± 4.29	10.95	49.80 ± 1.32	45.60 ± 1.19	28.61 ± 1.35	>250
	Inflorescence	8.07	69.74 ± 1.02	59.46 ± 1.18	44.77 ± 8.61	116.95 ± 3.56	8.8	25.78 ± 2.06	n.q.	n.q.	>300
*Tussilago farfara* L.	Leaf	5.68	70.76 ± 8.87	44.61 ± 6.98	31.65 ± 3.06	160.83 ± 4.25	10	80.25 ± 13.78	37.47 ± 7.13	34.95 ± 1.18	178.92 ± 8.71
***Crassulaceae***											
*Hylotelephium maximum*	Aerial part	20.94	<10	n.q.	n.q.	n.d.	4.35	44.85 ± 4.70			238.76 ± 1.68
***Equisetaceae***											
*Equisetum arvense* L.	Sterile stem	20.62	84.72 ± 9.20	60.95 ± 6.67	50.53 ± 3.80	62.16 ± 2.35	17.65	62.59 ± 9.02	59.85 ± 1.99	42.73 ± 1.56	155.61 ± 7.52
*Equisetum telmateia* L	Sterile stem	13.06	84.79 ± 1.97	74.61 ± 6.14	48.21 ± 1.33	63.93 ± 1.88	15.52	42.90 ± 6.41	25.18 ± 6.62	19.49 ± 5.71	>300
***Euphorbiaceae***											
*Euphorbia characias*	Aerial part	17.29	<10	n.q.	n.q.	n.d.	8.93	44.25 ± 5.01	32.03 ± 7.09	27.14 ± 6.72	>300
*Euphorbia helioscopia*	Aerial part	16.26	<10	n.q.	n.q.	n.d.	6.06	78.74 ± 4.15	68.74 ± 4.15	56.24 ± 5.01	41.95 ± 0.69
***Lamiaceae***											
*Calamintha sylavatica* Bromf ssp. *ascendens* (Jordan) P.W.Ball	Aerial part	12.27	64.58 ± 3.39	51.835.17	27.13 ± 2.20	67.90 ± 2.57	9.03	68.64 ± 2.06	28.58 ± 3.26	27.15 ± 3.53	157.84 ± 3.06
*Lavandula latifolia* Medicus	Inflorescence	13.38	98.73 ± 5.00	91.73 ± 9.67	66.13 ± 6.52	47.95 ± 0.59	8.76	91.25 ± 13.14	59.13 ± 1.90	72.18 ± 8.27	19.98 ± 0.49
	Stem and leaf	12.65	99.79 ± 4.00	98.27 ± 1.97	23.32 ± 2.59	70.92 ± 0.20	3.55	96.32 ± 2.71	63.07 ± 5.53	33.75 ± 1.70	71.92 ± 2.47
*Melissa officinalis* L. ssp. *officinalis*	Aerial part	6.79	61.81 ± 1.52	58.55 ± 16.81	48.99 ± 3.32	84.91 ± 2.16	11.82	59.40 ± 1.12	45.52 ± 3.58	23.81 ± 3.77	171.82 ± 4.25
*Mentha aquatica* L.	Aerial part	14.40	<10	n.q.	n.q.	n.d.	9.89	61.81 ± 1.25	55.00 ± 0.70	29.47 ± 3.56	118.87 ± 7.32
*Mentha longifolia* (L.) Hudson	Aerial part	9.45	77.98 ± 2.18	44.47 ± 2.40	30.18 ± 1.78	130.86 ± 7.22	6.44	90.45 ± 5.35	64.74 ± 1.00	44.08 ± 5.27	62.93 ± 1.68
*Mentha pullegium* L.	Aerial part	10.84	<10	n.q.	n.q.	n.d.	5.88	46.29 ± 1.40	38.91 ± 4.95	30.79 ± 1.34	226.7 ±10.98
*Mentha suaveolens* Ehrh.	Aerial part	10.34	62.29 ± 8.91	47.37 ± 5.81	34.01 ± 8.04	217.78 ± 11.48	8.61	57.74 ± 5.60	55.73 ± 7.08	47.91 ± 2.85	113.88 ± 3.96
*Origanum vulgare* L. spp. *virens* Bonnier and Layens	Inflorescence	8.50	61.89 ± 4.11	59.38 ± 2.10	56.25 ± 3.92	56.55 ± 0.62	6.54	62.91 ± 8.22	50.76 ± 2.15	43.54 ± 1.37	120.85 ± 1.88
	Aerial part	14.0	91.75 ± 1.38	80.21 ± 2.88	64.77 ± 5.35	4.62 ± 0.01	8.38	32.50 ± 3.30	18.07 ± 7.34	16.38 ± 1.09	>250
*Origanum vulgare* L. spp. *vulgare*	Aerial part	8.13	95.61 ± 2.02	91.50 ± 3.01	75.45 ± 2.92	2.59 ± 0.01	13.91	52.55 ± 7.86	44.35 ± 9.51	32.65 ± 1.03	175.82 ± 5.84
*Phlomis herba-venti* L.	Aerial part	13.54	72.47 ± 6.34	47.82 ± 5.14	48.57 ± 6.52	189.1 ± 2.67	6.16	62.84 ± 6.20	47.66 ± 4.52	42.84 ± 7.91	190.90 ± 4.15
*Phlomis lychnitis* L.	Inflorescence	14.52	53.63 ± 13.75	30.21 ± 2.90	39.33 ± 9.94	248.73 ± 5.64	6.22	<10	n.q.	n.q.	n.d.
	Stem and leaf	7.90	55.26 ± 5.04	28.27 ± 4.63	21.65 ± 4.53	247.75 ± 6.13	4.79	<10	n.q.	n.q.	n.d.
*Prunella vulgaris* L.	Aerial part	6.15	78.86 ± 7.39	68.78 ± 1.86	63.05 ± 3.11	24.97 ± 1.48	15.7	<10	n.q.	n.q.	n.d.
*Salvia pratensis* L.	Aerial part	9.99	41.17 ± 6.50	37.20 ± 5.14	21.86 ± 2.26	>300	13.95	<10	n.q.	n.q.	n.d.
*Sideritis hirsuta*	Aerial part	13.44	<10	n.q.	n.q.	n.d.	11.32	93.27 ± 3.85	87.40 ± 9.28	74.68 ± 3.67	31.96 ± 0.39
*Sideritis hyssopifolia* ssp. *guillonii*	Aerial part	3.44	<10	n.q.	n.q.	n.d.	4.83	47.45 ± 6.64	30.94 ± 1.06	28.33 ± 6.44	>250
*Teucrium chamaedrys*	Aerial part	11.01	98.09 ± 1.10	94.58 ± 11.84	69.98 ± 1.49	33.96 ± 0.19	7.45	98.96 ± 9.85	59.00 ± 8.80	41.05 ± 1.01	97.90 ± 1.88
*Thymus praecox* Opiz ssp. *polytrichus*	Aerial part	10.17	97.81 ± 10.9	60.16 ± 4.26	60.79 ± 5.39	71.92 ± 3.07	11.08	51.03 ± 1.16	33.05 ± 7.95	25.79 ± 3.04	>250
*Thymus vulgaris* L. ssp. *vulgaris*	Aerial part	5.09	82.48 ± 9.05	68.62 ± 8.91	45.47 ± 2.43	79.92 ± 3.36	9.64	<10	n.q.	n.q.	n.d.
***Lytraceae***											
*Lythrum salicaria* L.	Aerial part	20.04	<10	n.q.	n.q.	n.d.	4.07	98.50 ± 13.50	74.28 ± 6.04	48.62 ± 9.31	69.93 ± 1.48
***Papaveraceae***											
*Papaver rhoeas* L.	Capsule/petal	29.65	99.78 ± 7.57	56.02 ± 9.88	14.80 ± 1.57	76.92 ± 8.51	15.13	52.64 ± 5.31	42.64 ± 8.77	36.42 ± 1.40	150.84 ± 9.70
***Primulaceae***											
*Anagallis arvensis* L.	Aerial part	14.06	63.10 ± 1.08	57.92 ± 4.57	51.42 ± 5.56	57.94 ± 3.36	13.04	80.02 ± 1.25	58.98 ± 9.41	38.08 ± 8.33	102.89 ± 2.97
***Verbenaceae***											
*Verbena officinalis* L.	Aerial part	10.94	58.04 ± 7.63	43.20 ± 3.17	28.40 ± 5.13	166.83 ± 1.78	8.80	58.05 ± 1.61	39.96 ± 1.24	33.54 ± 1.85	140.85 ± 6.73
Galantamine			91.33 ± 1.31	88.38 ± 2.23	74.26 ± 6.20	19.9 ± 4.80					

n.d.–not determined; n.q.–not quantified.

**Table 2:** Spectrometric data, identification and molecular formula of phenolic constituents of ethanolic extract from *Origanum vulgare* ssp. *vulgare* aerial part.

Compound	Rt (min)	λ_max_ (nm)	[M–H]^–^ (*m/z*)	Fragment Ions (*m/z*)	Tentative Identification	Molecular Formula
**1**	1.1	296 sh, 324	179.05	135.04, 89.03	Caffeic acid (3,4-Dihydroxycinnamic acid)	C_9_H_8_O_4_
**2**	0.9	287 sh, 331	341.07	179.03, 149.04, 96.95	Caffeic acid 4-α-glucoside	C_15_H_18_O_9_
**3**	1.3	287 sh, 329	353.10	191.01, 179.03	Chlorogenic acid	C_16_H_18_O_9_
**4**	1.4	287 sh, 329	879.05	717, 1^-^, 179.05, 96.95	Caffeic acid tetramer glucoside (Rabdosiin 7-*O*-β-glucoside)	C_42_H_40_O_21_
**5**	2.4	254.6 348.5	609.17	463.3, 301.80	Rutin	C_27_H_30_O_16_
**6**	2.5	253, 290 sh, 370	629.13	477,03, 315.06, 96.95	Isorhamnetin 3-(6″-galloylglucoside)	C_29_H_26_O_16_
**7**	3.4	220.5 sh, 278.3	197.03	–	Syringic acid	C_9_H_10_O_5_
**8**	3.7	213.4 sh, 280.7	359.08	197.04	Syringic acid-4-β-glucoside	C_15_H_20_O_10_
**9**	5.6	269, 290 sh, 355	387.15	301.80	Quercetin oxalate	C_17_H_8_O_11_
**10**	5.8	266, 346	739.05	659.07, 593.3, 447.01, 285.03	Kaempferol-3-*O*-galactoside-6″-*O*-rhamnoside-3′″-*O*-rhamnoside	C_33_H_40_O_19_
**11**	6.3	289.0, 323.1 sh	537.09	493.11, 358.06, 295.06, 253.04, 185.02, 179.04, 135.04	Salvianolic acid H or Salvianolic I	C_27_H_22_O_12_
**12**	6.5	217.0, 261.7, 294.9	153.01	109.02	3,4-Dihydroxybenzoic acid (Protocatechuic acid)	C_7_H_6_O_4_
**13**	6.6	285, 325	449.19	377.04, 287.05 153.01	Eriodictyol-7-*O*-glucoside	C_21_H_22_O_11_
**14**	6.8	289.0, 323.1 sh	717.12	553.08, 519.09, 419.21, 358.06, 339.05, 321.04, 295.06, 179.04	Salvianolic acid D	C_36_H_30_O_16_
**15**	7.1	289.0, 323.1 sh	717.12	519.09, 421.1, 358.06, 339.05, 321.04, 179.04	Salvianolic acid B	C_36_H_30_O_16_
**16**	7.3	289.0, 323.1 sh	987.22	451.11, 179.04	Caffeic acid hexamer	C_52_H_44_O_20_
**17**	7.4	329.1	359.06	197,1, 179.05, 161.3, 135.04, 133.03, 123.04	Rosmarinic acid	C_18_H_16_O_8_
**18**	7.6	289.0, 323.1 sh	717.12	553.08, 519.09, 419.21, 358.06, 339.05, 321.04, 185.02, 179.04	Salvianolic acid L	C_36_H_30_O_16_
**19**	7.8	254,4	149.1-	-	Thymol	C_10_H_14_O
**20**	7.9	350, 268	357.06	357.09, 342.12, 327.07, 312.02, 297.02	Retusin	C_19_H_18_O_7_
**21**	8.0	254.6 348.5	653.14	507.4, 345.07, 330.1, 315.2, 96.95	Syringetin 3-*O*-rutinoside	C_29_H_34_O_17_
**22**	9.2	254, 267	447.05	357.78, 327.21, 285.4	Orientin (Luteolin 8-*C*-glucoside)	C_21_H_20_O_11_
**23**	9.4	254, 267	447.09	357.78, 327.22, 285.4	Homoorientin (Luteolin 6-*C*-glucoside)	C_21_H_20_O_11_

**Table 3:** Antioxidant activity against DPPH radical of ethanolic extract of *Origanum vulgare* ssp. *vulgare*. Results are expressed as percentage of inhibition (%), IC50 (µg/mL) values and activity index (AAI).

	Time (min)					
[Extract] (µg/mL)	15	30	45	60	75	90
125	105.29 ± 0.65	105.21 ± 0.64	105.35 ± 0.59	105.51 ± 0.71	105.49 ± 0.64	105.71 ± 0.67
62.5	104.89 ± 0.77	105.14 ± 0.59	105.16 ± 0.45	105.41 ± 0.71	105.28 ± 0.65	105.43 ± 0.67
31.25	102.98 ± 1.83	104.36 ± 0.85	104.71 ± 0.73	105.21 ± 0.89	105.28 ± 0.68	105.50 ± 0.64
15.62	90.40 ± 8.90	100.97 ± 6.86	103.29 ± 7.71	105.23 ± 6.09	106.00 ± 6.18	106.37 ± 5.29
7.81	71.11 ± 1.38	67.81 ± 6.37	72.48 ± 7.36	77.24± 6.10	78.11 ± 8.20	80.10 ± 8.31
3.91	50.46 ± 1.81	51.00 ± 2.27	51.02 ± 1.31	50.90 ± 1.26	49.53 ± 1.18	50.19 ± 1.41
1.95	36.66 ± 0.94	36.27 ± 0.76	38.89± 1.23	38.91 ± 1.45	41.78 ± 2.17	35.37 ± 1.68
0.98	19.45 ± 0.87	22.52 ± 2.46	21.71± 1.18	29.01 ± 0.80	29.26± 0.88	28.00 ± 1.32
**IC50 (µg/mL)**	4.05 ± 0.22 ^b^	3.82 ± 0.27 ^b^	3.58 ± 0.38 ^b^	3.22 ± 0.19 ^a^	3.15 ± 0.34 ^a^	3.28 ± 0.29 ^a^
**AAI**	4.94 ± 0.09 ^b^	5.23 ± 0.07 ^b^	5.59 ± 0.05 ^b^	6.21 ± 0.10 ^a^	6.35 ± 0.06 ^a^	6.10 ± 0.07 ^a^

Data expressed as means ± SD of triplicate analysis. Values with different letter present significant differences (*p* < 0.05) and same letter indicates no significant differences (*p* > 0.05). Value in bold means IC50_max_ (stabilization point).

**Table 4:** Pearson correlation coefficients between the AChE inhibition and antioxidant activity and the main compound content values.

	DPPH-AAI	ABTS	TPC	FL	DHBA	DHCA	SRA	SALVA	RA
AChE-IC50	−0.8649	−0.9487	−0.5984	−0.9563	−0.9247	−0.7667	−0.9864	−0.8806	−0.7693
DPPH-AAI		0.9378	0.8145	0.9141	0.9324	0.9011	0.9409	0.9208	0.9022
ABTS			0.8210	0.9878	0.9762	0.9304	0.9976	0.9923	0.9318
TPC				0.7220	0.9253	0.9732	0.8584	0.7439	0.9722
FL					0.9304	0.8618	0.9747	0.9995	0.8639
DHBA						0.9877	0.9888	0.9417	0.9884
DHCA							0.9534	0.8777	1.0000
SRA								0.9814	0.9546
SALVA									0.8796

AChE-IC50–acetylcholinesterase inhibition (IC50 µg/mL); DPPH-AAI–antioxidant activity index; ABTS (mg TE/100 mg extract); TPC–total phenolic compounds (mg/100 mg); FL–flavonoids (mg /100 mg); DHBA–dihydroxybenzoic acids (mg/100 mg); DHCA–dihydroxycinnamic acids (mg/100 mg); SRA–syringic acids (mg/100 mg); SALVA–salvianolic acids (mg/100 mg); RA–rosmarinic acid (mg RA/100 mg).

## Data Availability

The data presented in this study are available on request from the corresponding author.

## Sample Availability

Samples of the extracts are available available from the authors.

## References

[1] Breijyeh Z., Karaman R. (2020). Comprehensive Review on Alzheimer’s Disease: Causes and Treatment. Molecules.

[2] Uriarte-Pueyo I., Calvo M.I. (2011). Flavonoids as Acetylcholinesterase Inhibitors. Curr. Med. Chem..

[3] Yiannopoulou K.G., Papageorgiou S.G. (2013). Current and future treatments for Alzheimer’s disease. Ther. Adv. Neurol. Disord..

[4] Gnanaraj C., Sekar M., Fuloria S., Swain S.S., Gan S.H., Chidambaram K., Rani N.N.I.M., Balan T., Stephenie S., Lum P.T. (2022). In Silico Molecular Docking Analysis of Karanjin against Alzheimer’s and Parkinson’s Diseases as a Potential Natural Lead Molecule for New Drug Design, Development and Therapy. Molecules.

[5] Süntar I. (2020). Importance of ethnopharmacological studies in drug discovery: Role of medicinal plants. Phytochem. Rev..

[6] Calvo M.I., Cavero R.Y. (2015). Medicinal plants used for neurological and mental disorders in Navarra and their validation from official sources. J. Ethnopharmacol..

[7] Santos T.C., Gomes T.M., Pinto B.A.S., Camara A.L., Paes A.M.A. (2018). Naturally Occurring Acetylcholinesterase Inhibitors and Their Potential Use for Alzheimer’s Disease Therapy. Front. Pharmacol..

[8] Marston A., Kissling J., Hostettmann K. (2002). A rapid TLC bioautographic method for the detection of acetylcholinesterase and butyrylcholinesterase inhibitors in plants. Phytochem. Anal..

[9] López V., Akerreta S., Casanova E., García-Mina J.M., Cavero R.Y., Calvo M.I. (2007). In vitro antioxidant and anti-rhizopus activities of *Lamiaceae* herbal extracts. Plant Food. Hum. Nutr..

[10] López V., Akerreta S., Casasnova E., García-Mina J.M., Cavero R.Y., Calvo M.I. (2008). Screening of Spanish medicinal plants for antioxidant and antifungal activities. Pharm. Biol..

[11] Uysal S., Senkardes I., Mollica A., Zengin G., Bulut G., Dogan A., Glamočlija J., Soković M., Lobine D., Mahomoodally F.M. (2019). Biologically active compounds from two members of the *Asteraceae* family: *Tragopogon dubius* Scop. and *Tussilago farfara* L.. J. Biomol. Struct. Dyn..

[12] Seo S.M., Kim J., Kang J., Koh S.H., Ahn Y.J., Kang K.S., Park I.K. (2014). Fumigant toxicity and acetylcholinesterase inhibitory activity of 4 *Asteraceae* plant essential oils and their constituents against Japanese termite (*Reticulitermes speratus* Kolbe). Pestic. Biochem. Phys..

[13] Rodrigues A.M., Fale P.L., Ascensao L., Serralheiro M.L. (2014). *Santolina**impressa*, a Portuguese endemic species: Inhibition of acetylcholinesterase, antioxidant activity and cell toxicity. Planta Med..

[14] Gomes A., Pimpão R.C., Fortalezas S., Figueira I., Miguel C., Ferreira R.B., Santos C.N., Aguiar C., Salgueiro L., Cavaleiro C. (2015). Chemical characterization and bioactivity of phytochemicals from Iberian endemic *Santolina*
*semidentata* and strategies for ex situ propagation. Ind. Crop. Prod..

[15] Vladimir-Knežević S., Blažeković B., Kindl M., Vladić J., Lower-Nedza A.D., Brantner A.H. (2014). Acetylcholinesterase inhibitory, antioxidant and phytochemical properties of selected medicinal plants of the *Lamiaceae* family. Molecules.

[16] Truzzi E., Chaouch M.A., Rossi G., Tagliazucchi L., Bertelli D., Benvenuti S. (2022). Characterization and Valorization of the Agricultural Waste Obtained from *Lavandula* Steam Distillation for Its Reuse in the Food and Pharmaceutical Fields. Molecules.

[17] Videira R., Castanheira P., Graos M., Salgueiro L., Faro C., Cavaleiro C.A. (2013). Necrodane monoterpenoid from *Lavandula*
*luisieri* essential oil as a cell-permeable inhibitor of BACE-1, the beta-secretase in Alzheimer’s disease. Flavour Frag. J..

[18] Costa P., Gonalves S., Valentào P., Andrade P.B., Almeida C., Nogueira J.M.F., Romano A. (2013). Metabolic profile and biological activities of *Lavandula*
*pedunculata* subsp. lusitanica (Chaytor) Franco: Studies on the essential oil and polar extracts. Food Chem..

[19] Sebai H., Selmi S., Rtibi K., Gharbi N., Sakly M. (2015). Protective Effect of *Lavandula*
*stoechas* and *Rosmarinus officinalis* Essential Oils Against Reproductive Damage and Oxidative Stress in Alloxan-Induced Diabetic Rats. J. Med. Food.

[20] Costa P., Grevenstuk T., Rosa da Costa A.M., Gonçalves S., Romano A. (2014). Antioxidant and anti-cholinesterase activities of *Lavandula*
*viridis* L’Hér extracts after in vitro gastrointestinal digestion. Ind. Crop. Prod..

[21] Al-Sarar A.S., Hussein H.I., Abobakr Y., Bayoumi A.E., Al-Otaibi M.T. (2014). Fumigant toxicity and antiacetylcholinesterase activity of Saudi *Mentha longifolia* and *Lavandula dentata* species against *Callosobruchus maculatus* (F.) (Coleoptera: *Bruchidae*). Turk. Entomol. Derg..

[22] Stafford G.I., Pedersen M.E., van Staden J., Jäger A.K. (2008). Review on plants with CNS-effects used in traditional South African medicine against mental diseases. J. Ethnopharmacol..

[23] Lou H.Y., Fan P.H., Perez R.G., Lou H.X. (2011). Neuroprotective effects of linarin through activation of the PI3K/Akt pathway in amyloid-beta-induced neuronal cell death. Bioorgan. Med. Chem..

[24] Miyazawa M., Watanabe H., Kameoka H., Umemoto K. (1998). Inhibition of Acetylcholinesterase Activity by Essential Oils of *Mentha* Species. J. Agr. Food Chem..

[25] de Sousa Barros A., de Morais S.M., Ferreira P.A.T., Vieira Í.G.P., Craveiro A.A., dos Santos Fontenelle R.O., de Menezes J.E.S.A., da Silva F.W.F., de Sousa H.A. (2015). Chemical composition and functional properties of essential oils from *Mentha* species. Ind. Crop. Prod..

[26] Politeo O., Bektašević M., Carev I., Jurin M., Roje M. (2018). Phytochemical Composition, Antioxidant Potential and Cholinesterase Inhibition Potential of Extracts from *Mentha pulegium* L.. Chem. Biodivers..

[27] Ferreira A., Proença C., Serralheiro M.L.M., Araújo M.E.M. (2006). The in vitro screening for acetylcholinesterase inhibition and antioxidant activity of medicinal plants from Portugal. J. Ethnopharmacol..

[28] Sarikurkcu C., Zengin G., Oskay M., Uysal S., Ceylan R., Aktumsek A. (2015). Composition, antioxidant, antimicrobial and enzyme inhibition activities of two *Origanum vulgare* subspecies (subsp. vulgare and subsp. hirtum) essential oils. Ind. Crop. Prod..

[29] Yoo K.Y., Park S.Y. (2012). Terpenoids as Potential Anti-Alzheimer’s Disease Therapeutics. Molecules.

[30] López V., Cascella M., Benelli G., Maggi F., Gómez-Rincón C. (2018). Green drugs in the fight against Anisakis simplex-larvicidal activity and acetylcholinesterase inhibition of *Origanum compactum* essential oil. Parasitol. Res..

[31] López V., Pavela R., Gómez-Rincón C., Les F., Bartolucci F., Galiffa V., Petrelli R., Cappellacci L., Maggi F., Canale A. (2019). Efficacy of *Origanum syriacum* Essential Oil against the Mosquito Vector *Culex quinquefasciatus* and the Gastrointestinal Parasite Anisakis simplex, with Insights on Acetylcholinesterase Inhibition. Molecules.

[32] Loizzo M.R., Menichini F., Conforti F., Tundis R., Bonesi M., Saab A.M., Statti G.A., Cindio B., Houghton P.J., Menichini F. (2009). Chemical analysis, antioxidant, antiinflammatory and anticholinesterase activities of *Origanum ehrenbergii* Boiss and *Origanum syriacum* L. essential oils. Food Chem..

[33] Park S.J., Kim D.H., Lee I.K., Jung W.Y., Park D.H., Kim J.M., Lee K.R., Lee K.T., Shin C.Y., Cheong J.H. (2010). The ameliorating effect of the extract of the flower of *Prunella*
*vulgaris* var. lilacina on drug-induced memory impairments in mice. Food Chem. Toxicol..

[34] Qu Z., Zhang J., Yang H., Gao J., Chen H., Liu C., Gao W. (2017). *Prunella vulgaris* L., an Edible and Medicinal Plant, Attenuates Scopolamine-Induced Memory Impairment in Rats. J. Agric. Food Chem..

[35] Miguel M., Bouchmaaa N., Aazza S., Gaamoussi F., Lyoussi B. (2014). Antioxidant, anti-inflammatory and anti-acetylcholinesterase activities of eleven extracts of Moroccan plants. Fresen. Environ. Bull..

[36] Topcu G., Ertas A., Ozturk M., Dincel D., Kilic T., Halfon B. (2011). Ent-kaurane diterpenoids isolated from *Sideritis*
*congesta*. Phytochem. Lett..

[37] Erdogan-Orhan I., Baki E., Şenol S., Yilmaz G. (2010). Sage-called plant species sold in Turkey and their antioxidant activities. J. Serb. Chem. Soc..

[38] Golfakhrabadi F., Yousefbeyk F., Mirnezami T., Khanavi M., Laghaei P., Hajimahmoodi M. (2015). Antioxidant and Antiacetylcholinesterase Activity of *Teucrium*hyrcanicum. Pharmacog. Res..

[39] Ahmad B., Mukarram Shah S.M., Khan H., Hassan Shah S.M. (2007). Enzyme inhibition activities of *Teucrium*
*royleanum*. J. Enzyme Inhib. Med. Chem..

[40] Rabiei Z., Mokhtari S., Asgharzade S., Rahnama S., Rafieian-kopaei M., Gholami M. (2015). Inhibitory effect of *Thymus*
*vulgaris* extract on memory impairment induced by scopolamine in rat. Asian Pac. J. Trop. Biomed..

[41] Sezer Senol F., Orhan I.E., Ozgen U., Renda G., Bulut G., Guven L., Karaoglan E.S., Sevindik H.G., Skalicka-Wozniak K., Koca Caliskan U. (2016). Memory-vitalizing effect of twenty-five medicinal and edible plants and their isolated compounds. S. Afr. J. Bot..

[42] Singh D.K., Agarwal R.A. (1987). Latex of *Euphorbia*
*antisyphlitica*, a new potent molluscicide having antiacetylcholinesterase activity against the snail *Lymnaea acuminata*. Sci. Total Environ..

[43] Pisano M.B., Cosentino S., Viale S., Spanò D., Corona A., Esposito F., Tramontano E., Montoro P., Tuberoso C.I., Medda R. (2016). Biological Activities of Aerial Parts Extracts of *Euphorbia characias*. Biomed. Res. Int..

[44] Anuradha H., Srikumar B.N., Deepti N., Shankaranarayana Rao B.S., Lakshmana M. (2010). Restoration of acetylcholinesterase activity by *Euphorbia*
*hirta* in discrete brain regions of chronically stressed rats. Pharm. Biol..

[45] Tiwari S., Singh A. (2004). Piscicidal and anti-acetylcholinesterase activity of *Euphorbia*
*royleana* stem bark extracts against freshwater common predatory fish *Channa punctatus*. Environ. Toxicol. Phar..

[46] Bakry F.A. (2009). Use of some plant extracts to control *Biomphalaria alexandrina* snails with emphasis on some biological effects. Pestic. Biochem. Phys..

[47] Tiwari S., Singh A. (2006). Biochemical stress response in freshwater fish *Channa punctatus* induced by aqueous extracts of *Euphorbia tirucalli* plant. Chemosphere.

[48] Wei J.C., Zhang X.Y., Gao Y.N., Wang D.D., He X.L., Gao X.X., Hu G.S., Wang A.H., JJia J.M. (2021). Euphorfinoids E-L: Diterpenoids from the roots of *Euphorbia fischeriana* with acetylcholinesterase inhibitory activity. Phytochemistry.

[49] Kim D. (2002). Inhibitory effect of corynoline isolated from the aerial parts of *Corydalis incisa* on the acetylcholinesterase. Arch. Pharm. Res..

[50] Gayoso L., Roxo M., Cavero R.Y., Calvo M.I., Ansorena D., Astiasarán I., Wink M. (2018). Bioaccessibility and biological activity of *Melissa officinalis*, *Lavandula latifolia* and *Origanum vulgare* extracts: Influence of an in vitro gastrointestinal digestion. J. Funct. Foods.

[51] Mahomoodally M.F., Zengin G., Aladag M.O., Ozparlak H., Diuzheva A., Jekő J., Cziáky Z., Aumeeruddy M.Z. (2019). HPLC-MS/MS chemical characterization and biological properties of *Origanum onites* extracts: A recent insight. Int. J. Environ. Health Res..

[52] Wagner H., Bladt S. (1996). Plant Drug Analysis.

[53] Gonçalves S., Moreira E., Grosso C., Andrade P.B., Valentão P., Romano A. (2017). Phenolic profile, antioxidant activity and enzyme inhibitory activities of extracts from aromatic plants used in Mediterranean diet. J. Food Sci. Technol..

[54] Oniga I., Pușcaș C., Silaghi-Dumitrescu R., Olah N.K., Sevastre B., Marica R., Marcus I., Sevastre-Berghian A.C., Benedec D., Pop C.E. (2018). *Origanum vulgare* ssp. vulgare: Chemical composition and biological studies. Molecules.

[55] Martins N., Barros L., Santos-Buelga C., Henriques M., Silva S., Ferreira I.C.F.R. (2014). Decoction, infusion and hydroalcoholic extract of *Origanum vulgare* L.: Different performances regarding bioactivity and phenolic compounds. Food Chem..

[56] Lin Y.L., Wang C.N., Shiao Y.J., Liu T.Y., Wang W.Y. (2003). Benzolignanoid and polyphenols from *Origanum vulgare*. J. Chin. Chem. Soc..

[57] Agiomyrgianaki A., Dais P. (2012). Simultaneous determination of phenolic compounds and triterpenic acids in oregano growing wild in Greece by 31P NMR spectroscopy. Magn. Reson. Chem..

[58] González M.D., Luis C.M., Lanzelotti P.L. (2014). Polyphenolic profile of *Origanum vulgare* L. ssp. viridulum from Argentina. Fyt. Issn..

[59] Koldaş S., Demirtas I., Ozen T., Demirci M.A., Behçet L. (2015). Phytochemical screening, anticancer and antioxidant activities of *Origanum vulgare* L. ssp. viride (Boiss.) Hayek, a plant of traditional usage. J. Sci. Food Agric..

[60] Skotti E., Anastasaki E., Kanellou G., Polissiou M., Tarantilis P.A. (2014). Total phenolic content, antioxidant activity and toxicity of aqueous extracts from selected Greek medicinal and aromatic plants. Ind. Crops Prod..

[61] Roby M.H.H., Sarhan M.A., Selim K.A.H., Khalel K.I. (2013). Evaluation of antioxidant activity, total phenols and phenolic compounds in thyme (*Thymus vulgaris* L.), sage (*Salvia officinalis* L.), and majoram (*Origanum majorana* L.) extracts. Ind. Crops Prod..

[62] Lin L.Z., Mukhopadhyay S., Robbins R.J., Harnly J.M. (2007). Identification and quantification of flavonoids of Mexican oregano (*Lippia graveolens*) by LC-DAD-ESI/MS analysis. J. Food Compost. Anal..

[63] Ličina B.Z., Stefanović O.D., Vasić S.M., Radojević I.D., Dekić M.S., Čomić L.R. (2013). Biological activities of the extracts from wild growing *Origanum vulgare* L.. Food Control.

[64] Hossain M.B., Rai D.K., Brunton N.P., Martin-Diana A.B., Barry-Ryan A.C. (2010). Characterization of phenolic composition in *Lamiaceae* spices by LC-ESI-MS/MS. J. Agric. Food Chem..

[65] de Torre M.P., Vizmanos J.L., Cavero R.Y., Calvo M.I. (2020). Improvement of antioxidant activity of oregano (*Origanum vulgare* L.) with an oral pharmaceutical form. Biomed. Pharmacother..

[66] Liu B., Hu T., Yan W. (2020). Authentication of the Bilberry Extracts by an HPLC Fingerprint Method Combining Reference Standard Extracts. Molecules.

[67] Jorge T.F., Mata A.T., António C. (2016). Mass spectrometry as a quantitative tool in plant metabolomics. Philos. Trans. A. Math. Phys. Eng. Sci..

[68] Hamasaki N., Ishii E., Tominaga K., Tezuka Y., Nagaoka T., Kadota S., Kuroki T., Yano I. (2000). Highly selective antibacterial activity of novel alkyl quinolone alkaloids from a Chinese herbal medicine, Gosyuyu (Wu-Chu-Yu), against *Helicobacter pylori* in vitro. Microbiol. Immunol..

[69] Lecomte J., López-Giraldo L.J., Laguerre M., Baréa B., Villeneuve P. (2010). Synthesis, Characterization and Free Radical Scavenging Properties of Rosmarinic Acid Fatty Esters. J. Am. Oil Chem. Soc..

[70] Petersen M., Abdullah Y., Benner J., Eberle D., Gehlen K., Hücherig S., Janiak V., Kim K.H., Sander M., Weitzel C. (2009). Evolution of rosmarinic acid biosynthesis. Phytochemistry.

[71] Gayoso L., Claerbout A.S., Calvo M.I., Cavero R.Y., Astiasarán I., Ansorena D. (2016). Bioaccessibility of rutin, caffeic acid and rosmarinic acid: Influence of the in vitro gastrointestinal digestion models. J. Funct. Foods.

[72] Agata I., Hatanp T., Okudaq T.A. (1989). Tetrameric derivative of caffeic acid from *Rabdosia japonica*. Phytochemistry.

[73] Flegkas A., Milosević Ifantis T., Barda C., Samara P., Tsitsilonis O., Skaltsa H. (2019). Antiproliferative Activity of (-)-Rabdosiin Isolated from *Ocimum sanctum* L.. Medicines.

[74] Teixeira B., Marques A., Ramos C., Serrano C., Matos O., Neng N.R., Nogueira J.M.F., Saraiva J.A., Nunes M.L. (2013). Chemical composition and bioactivity of different oregano (*Origanum vulgare*) extracts and essential oil. J. Sci. Food Agric..

[75] Liang C.H., Chan L.P., Ding H.Y., So E.C., Lin R.J., Wang H.M., Chen Y.G., Chou T.H. (2012). Free radical scavenging activity of 4-(3,4-Dihydroxybenzoyloxymethyl) phenyl-O-β-d-glucopyranoside from *Origanum vulgare* and its protection against oxidative damage. J. Agric. Food Chem..

[76] Zengin G., Ferrante C., Orlando G., Zheleva-Dimitrova D., Gevrenova R., Recinella L., Chiavaroli A., Leone S., Brunetti L., Aumeeruddy M.Z. (2019). Chemical profiling and pharmaco-toxicological activity of *Origanum sipyleum* extracts: Exploring for novel sources for potential therapeutic agents. J. Food Biochem..

[77] Li F., Tsona N.T., Li J., Du L. (2021). Aqueous-phase oxidation of syringic acid emitted from biomass burning: Formation of light-absorbing compounds. Sci. Total Environ..

[78] Li Y., Zhang L., Wang X., Wu W., Qin R. (2019). Effect of Syringic acid on antioxidant biomarkers and associated inflammatory markers in mice model of asthma. Drug Dev. Res..

[79] Taleb M.H., Abdeltawab N.F., Shamma R.N., Abdelgayed S.S., Mohamed S.S., Farag M.A., Ramadan M.A. (2018). *Origanum vulgare* L. essential oil as a potential anti-acne topical nanoemulsion-*in vitro* and in vivo study. Mol. A J. Synth. Chem. Nat. Prod. Chem..

[80] Llana-Ruiz-Cabello M., Gutiérrez-Praena D., Puerto M., Pichardo S., Jos Á., Cameán A.M. (2015). In vitro pro-oxidant/antioxidant role of carvacrol, thymol and their mixture in the intestinal Caco-2 cell line. Toxicol. Vitr..

[81] Gutiérrez-Grijalva E.P., Angulo-Escalante M.A., León-Félix J., Heredia J.B. (2017). Effect of in vitro digestion on the Total Antioxidant Capacity and Phenolic Content of 3 species of oregano (*Hedeoma patens*, *Lippia graveolens*, *Lippia palmeri*). J. Food Sci..

[82] Laothaweerungsawat N., Sirithunyalug J., Chaiyana W. (2020). Chemical compositions and anti-skin-ageing activities of *Origanum vulgare* L. essential oil from tropical and mediterranean region. Molecules.

[83] Zhang Q., Wang S., Yu Y., Sun S., Zhang Y., Zhang Y., Yang W., Li S., Qiao Y. (2016). Salvianolic Acid A, as a Novel ETA Receptor Antagonist, Shows Inhibitory Effects on Tumor in Vitro. Int. J. Mol. Sci..

[84] Chishti S., Kaloo Z.A., Sultan P. (2013). Medicinal importance of genus *Origanum*: A review. J. Pharmacogn. Phytotherapy.

[85] Gulcin I. (2020). Antioxidants and antioxidant methods: An updated overview. Arch. Toxicol..

[86] Zhang X.L., Guo Y.S., Wang C.H., Li G.Q., Xu J.J., Chung H.Y., Ye W.C., Li Y.L., Wang G.C. (2014). Phenolic compounds from *Origanum vulgare* and their antioxidant and antiviral activities. Food Chem..

[87] Gutiérrez-Grijalva E.P., Picos-Salas M.A., Leyva-López N., Criollo-Mendoza M.S., Vazquez-Olivo G., Heredia J.B. (2018). Flavonoids and phenolic acids from oregano: Occurrence, biological activity and health benefits. Plants.

[88] Baâtour O., Kaddour R., Mahmoudi H., Tarchoun I., Bettaieb I., Nasri N., Mrah S., Hamdaoui G., Lachaâl M., Marzouk B. (2012). Culture conditions and salt effects on essential oil composition of sweet marjoram (*Origanum majorana*) from Tunisia. Acta Pharm..

[89] De Falco E., Roscigno G., Landolfi S., Scandolera E., Senatore F. (2014). Growth, essential oil characterization, and antimicrobial activity of three wild biotypes of oregano under cultivation condition in Southern Italy. Ind. Crops Prod..

[90] Valko M., Leibfritz D., Moncol J., Cronin M.T.D., Mazur M., Telser J. (2007). Free radicals and antioxidants in normal physiological functions and human disease. Int. J. Biochem. Cell Biol..

[91] Viña J., Borras C., Abdelaziz K.M., Garcia–Valles R., Gomez-Cabrera M.C. (2013). The Free Radical Theory of Aging Revisited The Cell Signaling Disruption Theory of Aging. Antioxid. Redox Signal..

[92] Bajaj S., Khan A. (2012). Antioxidants and diabetes. Indian J. Endocrinol. Metab..

[93] Botsoglou N.A., Taitzoglou I.A., Botsoglou E., Lavrentiadou S.N., Kokoli A.N., Roubies N. (2008). Effect of Long-Term Dietary Administration of Oregano on the Alleviation of Carbon Tetrachloride-Induced Oxidative Stress in Rats. J. Agric. Food Chem..

[94] Nur Alam M., Jahan Bristi N., Rafiquzzaman M. (2013). Review on in vivo and in vitro methods evaluation of antioxidant activity. Saudi Pharm. J..

[95] Brand-Williams W., Cuvelier M.E., Berset C. (1995). Use of a free radical method to evaluate antioxidant activity. LWT–Food Sci. Technol..

[96] de Torre M.P., Cavero R.Y., Calvo M.I., Vizmanos J.L. (2019). A Simple and a Reliable Method to Quantify Antioxidant Activity *In Vivo*. Antioxidants.

[97] Scherer R., Godoy H.T. (2009). Antioxidant activity index (AAI) by the 2,2-diphenyl-1-picrylhydrazyl method. Food Chem..

[98] García-Herreros C., García-Iñiguez M., Astiasarán I., Ansorena D. (2010). Antioxidant activity and phenolic content of water extracts of *Borago officinalis* L.: Influence of plant part and cooking procedure. Ital. J. Food Sci..

[99] Orhan I.E., Kucukboyaci N., Calis I., Cerón-Carrasco H.P., den Haan Alonso H., Peña-García J., Pérez-Sánchez H. (2017). Acetylcholinesterase inhibitory assessment of isolated constituents from *Salsola grandis* Freitag, Vural & Adıgüzel and molecular modeling studies on N-acetyltryptophan. Phytochem. Lett..

[100] Khan H., Marya, Amin S., Kamal M.A., Patel S. (2018). Flavonoids as acetylcholinesterase inhibitors: Current therapeutic standing and future prospects. Biomed. Pharmacother..

[101] Szwajgier D. (2015). Anticholinesterase activity of selected phenolic acids and flavonoids—Interaction testing in model solutions. Ann. Agric. Environ. Med..

[102] Szwajgier D., Baranowska-Wójcik E., Borowiec K. (2018). Phenolic acids exert anticholinesterase and cognition-improving effects. Curr. Alzheimer Res..

[103] Srinivasulu C., Ramgopal M., Ramanjaneyulu G., Anuradha C.M., Suresh Kumar C. (2018). Syringic acid (SA)—A Review of Its Occurrence, Biosynthesis, Pharmacological and Industrial Importance. Biomed. Pharmacother..

[104] Budryn G., Majak I., Grzelczyk J., Szwajgier D., Rodríguez-Martínez A., Pérez-Sánchez H. (2022). Hydroxybenzoic Acids as Acetylcholinesterase Inhibitors: Calorimetric and Docking Simulation Studies. Nutrients.

[105] Uriarte-Pueyo I., Calvo M.I. (2010). Structure-activity relationships of acetylated flavone glycosides from *Galeopsis ladanum* L. (*Lamiaceae*). Food Chem..

[106] Rhee I.K., Meent M.V., Ingkaninan K., Verpoorte R. (2001). Screening for acetylcholinesterase inhibitors from *Amaryllidaceae* using silica gel thin-layer chromatography in combination with bioactivity staining. J. Chromatogr. A.

[107] Carpinella M.C., Andrione D.G., Ruiz G., Palacios S.M. (2010). Screening for acetylcholinesterase inhibitory activity in plant extracts from Argentina. Phytother. Res..

[108] Guedes L., Reis P.B.P.S., Machuqueiro M., Ressaissi A., Pacheco R., Serralheiro M.L. (2019). Bioactivities of *Centaurium erythraea* (*Gentianaceae*) Decoctions: Antioxidant activity, enzyme inhibition and docking studies. Molecules.

